# A Novel Lineage of Cile-Like Viruses Discloses the Phylogenetic Continuum Across the Family *Kitaviridae*

**DOI:** 10.3389/fmicb.2022.836076

**Published:** 2022-03-28

**Authors:** Pedro L. Ramos-González, Camila Chabi-Jesus, Aline D. Tassi, Renata Faier Calegario, Ricardo Harakava, Claudia F. Nome, Elliot W. Kitajima, Juliana Freitas-Astua

**Affiliations:** ^1^Laboratório de Biologia Molecular Aplicada, Instituto Biológico de São Paulo, São Paulo, Brazil; ^2^Escola Superior de Agricultura Luiz de Queiroz (ESALQ), Universidade de São Paulo, Piracicaba, Brazil; ^3^Departamento de Fitotecnia e Fitossanidade, Universidade Federal do Paraná, Curitiba, Brazil; ^4^Instituto de Patologia Vegetal, Centro de Investigaciones Agropecuarias, Instituto Nacional de Tecnología Agropecuaria (INTA), Córdoba, Argentina; ^5^Embrapa Mandioca e Fruticultura, Cruz das Almas, Brazil

**Keywords:** *Brevipalpus*-transmitted viruses, high-throughput sequencing, ornamental plants, *Brevipalpus* mites, virion morphology, viral mixed infections

## Abstract

An increasing number of plant species have been recognized or considered likely reservoirs of viruses transmitted by *Brevipalpus* mites. A tiny fraction of these viruses, primarily those causing severe economic burden to prominent crops, have been fully characterized. In this study, based on high-throughput sequencing, transmission electron microscopy analyses of virions in plant-infected tissues, viral transmission experiments, and the morphoanatomical identification of the involved *Brevipalpus* mites, we describe molecular and biological features of viruses representing three new tentative species of the family *Kitaviridae*. The genomes of Solanum violifolium ringspot virus (SvRSV, previously partially characterized), Ligustrum chlorotic spot virus (LigCSV), and Ligustrum leprosis virus (LigLV) have five open reading frames (ORFs) > 500 nts, two distributed in RNA1 and three in RNA2. RNA1 of these three viruses display the same genomic organization found in RNA1 of typical cileviruses, while their RNA2 are shorter, possessing only orthologs of genes *p61*, *p32*, and *p24.* LigCSV and LigLV are more closely related to each other than to SvRSV, but the identities between their genomic RNAs were lower than 70%. In gene-by-gene comparisons, ORFs from LigCSV and LigLV had the highest sequence identity values (nt sequences: 70–76% and deduced amino acid sequences: 74–83%). The next higher identity values were with ORFs from typical cileviruses, with values below 66%. Virions of LigLV (≈ 40 nm × 55 nm) and LigCSV (≈ 54 nm × 66 nm) appear almost spherical, contrasting with the bacilliform shape of SvRSV virions (≈ 47 nm × 101 nm). Mites collected from the virus-infected plants were identified as *Brevipalpus papayensis, B. tucuman*, and *B. obovatus.* Viruliferous *B. papayensis* mites successfully transmitted LigCSV to *Arabidopsis thaliana*. SvRSV, LigCSV, and LigLV seem to represent novel sub-lineages of kitaviruses that descent on parallel evolutionary branches from a common ancestor shared with the tentative cile-like virus hibiscus yellow blotch virus and typical cileviruses. Biological and molecular data, notably, the phylogenetic reconstruction based on the RdRp proteins in which strong support for monophyly of the family *Kitaviridae* is observed, mark an advance in the understanding of kitavirids.

## Introduction

The family *Kitaviridae*, order *Martellivirales*, groups plant-infecting viruses with linear single-stranded (ss) positive (+) split RNA genomes which are assigned to the genera *Cilevirus*, *Higrevirus*, or *Blunervirus* (Quito-Avila et al., 2021). Except for the blunervirus tea plant necrotic ring blotch virus (TPNRBV) (Hao et al., 2018), kitavirids stand out by the production of non-systemic diseases wherein only the locally infected tissues typically develop chlorotic and/or necrotic lesions (Freitas-Astúa et al., 2018; Quito-Avila et al., 2021). In the case of cileviruses, whose vectorial transmission by mites of the genus *Brevipalpus* has been long-established (Alberti and Kitajima, 2014; de Lillo et al., 2021), the viral spread remains restricted to tissues around the mite feeding sites. Cyto- and physiopathological effects ensuing from the infection by the cilevirus citrus leprosis C (CiLV-C) resemble a hypersensitive like-response (Marques et al., 2010; Arena et al., 2016, 2020).

Based on the sequences of the RNA-dependent RNA polymerase (RdRp) and P24 proteins, kitavirids display a close phylogenetic relationship with an increasing number of unclassified arthropod-infecting viruses including those belonging to the groups centivirus, aphiglyvirus, and negevirus (Kondo et al., 2020; Morozov et al., 2020; Ramos-González et al., 2020). Kitaviruses have quasi-spherical or bacilliform virions that in the case of cileviruses are enveloped particles (Kitajima and Alberti, 2014), a feature shared with negeviruses (Vasilakis et al., 2013).

The genome of typical cileviruses is split into two molecules in which RNA1, ≈9 kb, comprises two open reading frames (ORFs), *RdRp* and *p29*, whereas RNA2, ≈5 kb, includes four canonical ORFs (*p15*, *p61*, *p32*, and *p24*) (Freitas-Astúa et al., 2018). The 5′-end of the cilevirus RNA2 is of variable length and organization (Ramos-González et al., 2021). Between *p15* and *p61*, or overlapping *p15*, cilevirus genomes harbor orphan ORFs which in some cases encode predicted small proteins with predicted transmembrane domains (TM) (Ramos-González et al., 2020, 2021). Hibiscus yellow blotch virus (HYBV), a cile-like virus infecting hibiscus plants in Hawaii, has a genomic organization that differs from those commonly observed in typical cileviruses i.e., CiLV-C, citrus leprosis virus C2 (CiLV-C2), and passion fruit green spot virus (PfGSV) (Locali-Fabris et al., 2006; Roy et al., 2013; Ramos-González et al., 2020; Olmedo-Velarde et al., 2021). Compared with typical cileviruses, HYBV lacks the genomic region upstream of the ORF *p61*, while its ORF ortholog of *p29* is located at the 3′-end of the RNA2 instead of its regular locus at the 3′-end of RNA1.

A disease called “*lepra explosiva de la ligustrina*” (LEL) that affects privet (*Ligustrum sinense*), an ornamental shrub, was first observed at the end of the 1930s, in Argentina (Vergani, 1942). LEL drew attention because of the similarity between its symptoms in leaves and twigs and those generated by “*lepra explosiva*,” a serious disease of citrus concomitantly described in Argentina, Brazil, and Paraguay later referred to as citrus leprosis and caused by a range of cileviruses and dichorhaviruses (Bitancourt, 1940, 1955; Frezzi, 1940; Vergani, 1942; Bastianel et al., 2010; Ramos-González et al., 2018). Affected privet leaves showed isolated circular or irregular spots with 2–4 mm in diameter (sometimes reaching 6–8 mm in diameter) with yellowish-green halos, in which the center of the lesion was usually noticeably chlorotic. The transmission of LEL was first demonstrated to be carried out by *Tenuipalpus pseudocuneatus* Blanchard, a mites species later synonymized with *Brevipalpus obovatus* (Welbourn et al., 2003). The application of miticides successfully blocked the transmission of LEL to both new healthy plants and fresh shoots of the diseased plants (Vergani, 1942). The putative causal agent of the disease was tentatively named Ligustrum leprosis virus (Tassi, 2018).

In 1976, shrubs of *Ligustrum lucidum* in a hedgerow in the Agricultural Science campus of Federal University of Paraná, in Curitiba, State of Paraná (PR), Brazil, were found affected by ring spots in the leaves (Lima Neto et al., 1994). Abundant aggregated bacilliform particles of approximately 28 × 103 nm inside enlarged cisternae in the perinuclear region and/or large electronluscent vesicles, likely viroplasms, associated with the rough endoplasmic reticulum were detected in the parenchyma cells. Based on particle morphology, which was somehow similar to those of rhabdoviruses but of incongruent size, and, particularly, the difference in the cytopathological effects observed in the infected cells, it was suggested the presence of a new type of virus (Lima et al., 1991; Lima Neto et al., 1994).

A tentative cilevirus called Solanum violifolium ringspot virus (SvRSV) was first described in Piracicaba, State of São Paulo (SP), Brazil, in 2007 (Ferreira et al., 2007). SvRSV-infected leaves of the ornamental creeping plant *Solanum violifolium* show chlorotic spots which can turn into necrotic lesions, and occasionally, when senescent, they can display green islands. Experimentally, SvRSV can be successfully transmitted to *Arabidopsis thaliana* and other plants of the families Solanaceae, Amaranthaceae, and Malvaceae, mechanically or by using viruliferous mites of the species *B*. *yothersi* (previously known as *Brevipalpus phoenicis* sensu lato) (Ferreira et al., 2007; Arena et al., 2017). The virus is also transmitted by *B. obovatus*, which seems to be its natural vector (Ferreira et al., 2007).

In SvRSV-infected plants, abundant enveloped bacilliform particles with 50–60 nm in width and an extremely heterogeneous length, 100–1,000 nm, were commonly observed in the lumen of the endoplasmic reticulum and vesicles likely derived from the endoplasmic reticulum (Ferreira et al., 2007). Cytopathic effects such as the presence of electron-dense areas were noticed in the cytoplasm of cells of the plant species *S. violifolium, Datura suaveolens*, *Salvia leucantha*, *Thumbergia erecta*, *Hibiscus cannabinus*, and *Capsicum annum*. A fragment of approximately 600 bp derived from the ORF encoding the RdRp of SvRSV was obtained from an infected *S. violifolium* plant collected in Piracicaba, SP, Brazil, in 2006 (GenBank accession number DQ514336). The fragment showed a relatively low nucleotide (nt) sequence identity (<62%) with the genome of CiLV-C (Ferreira et al., 2007). Using the same specific primer pair, SvRSV was detected in an infected *Gomesa bifolia* plant, a small, cool-temperature native orchid from South America collected in Córdoba, Argentina, in 2012 (GenBank accession number KT733671).

In this study, we describe the full-genomic sequences of SvRSV and two other related cile-like viruses found in several ornamental plants collected in Brazil and Argentina. Additionally, mites found on the leaf samples were identified by morphoanatomical evaluations and in some cases were used for viral transmission to arabidopsis plants. The size of virions detected in natural and experimental hosts was measured and compared with those of the typical cilevirus PfGSV. Finally, we mapped the distribution of two kitaviruses found infecting the same sample by detecting their RNA molecules in isolated leaf lesions.

## Materials and Methods

### Plant Material

**FIGURE 1:**
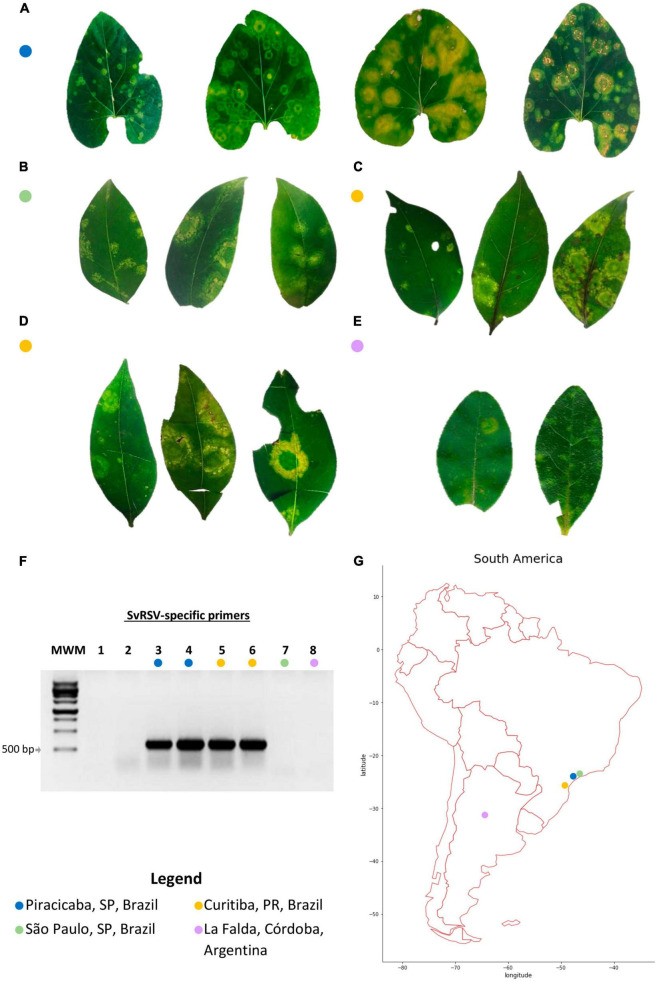
Leaf symptoms, detection of the partially characterized Solanum violifolium ringspot virus (SvRSV) in the RNA extracts, and place of collection of the samples. **(A)** Sample Prb1. Leaves of the ornamental herbaceous *Solanum violifolium* plant were likely collected during different stages of disease progression. The leaf on the left shows small chlorotic ring spots and was likely collected a few days after the infection. In contrast, the three leaves on the right show large chlorotic spots that also include necrotic areas in form of concentric circles. **(B)** Sample SPa1. Leaves of the ornamental shrub *Ligustrum japonicum*, collected in a private garden, show chlorotic spots with yellow irregular lesions, and regular concentric rings. **(C)** Sample Crb1. Leaves of *L. lucidum* were collected in the public garden *Bosque do Papa* in the city of Curitiba, PR, showing yellow concentric spots of variable sizes. **(D)** Sample Crb2. Leaves of *L. japonicum* plants were collected in a hedgerow in the Agricultural Science campus of the Federal University of Paraná. Typical symptoms consisted of large yellowish concentric spots and necrotic areas were infrequently observed. **(E)** Sample Cdb1. Leaves of *L. sinense* shrubs showing small (<0.5 cm in diameter) to medium (≈ 1 cm in diameter) light yellowish to greenish spots. The largest lesions showed small rings of necrotic tissues. **(F)** 1% agarose gel electrophoresis of RT-PCR products for the detection of the tentative cilevirus SvRSV using specific primers. The expected size of the amplicons is ≈ 600 bp. MWM: molecular weight marker, M1181 Ladder (Sinapse Biotechnology, São Paulo, SP, BR). Lanes 1 and 2: Reverse-transcription reaction and PCR negative controls, respectively. Lanes 3–8 correspond with samples as indicated by the colored circles; lanes 3 and 4: two RNA extracts from sample Prb1; 5: sample Crb1; 6: sample Crb2; 7: sample SPa1; and 8: sample Cdb1. **(G)** South America map indicating the place of collection of samples evaluated in this study.

Leaf samples from *Solanum violifolium* Schott plants showing chlorotic and necrotic spots were collected in Piracicaba, SP, Brazil, in 2014 (sample Prb1). Branches of privet shrubs of the species *Ligustrum lucidum, L. japonicum*, and *L. sinense* with leaves mainly showing chlorotic spots were collected in urban public gardens of the cities of São Paulo, SP, Brazil, in 2018 (sample SPa1); Curitiba, PR, Brazil, in 2018 (samples Crb1 and Crb2); and La Falda, Córdoba, Argentina, in 2019 (sample Cdb1), respectively. Pictures of all samples were taken immediately after their collection (Figure 1).

Plant samples were examined under a stereo-microscope (Leica M125, Wetzlar, Germany) or a hand-held magnifying glass to detect the presence of *Brevipalpus* mites and collect them when found. Mites were kept in 90% ethanol solution until their taxonomic identification. Small pieces of leaf lesions were removed using new razor blades or sterile scissors. Fragments of these tissues were prefixed in a modified Karnovsky solution (2.5% glutaraldehyde and 2% paraformaldehyde in 0.05 M cacodylate buffer, pH 7.2) (Kitajima and Nome, 1999) for transmission electron microscopy (TEM) analyses or conserved in RNA stabilization solution (RNA*later*, Thermo Fisher Scientific, Waltham, MA, United States) until RNA extraction.

All of the preserved biological materials and fresh leaves and branches of plants collected in Brazil were sent to Instituto Biológico de São Paulo, SP, Brazil, and Escola Superior de Agricultura Luiz de Queiroz, Piracicaba, SP, Brazil. Living mites were captured from fresh samples and used for virus transmission experiments. Symptomatic plant tissues from these samples were stored at -80°C until their use in molecular analyses. Healthy tissues from plants of each of the studied species were also collected and used as negative controls in reverse transcriptase-PCR viral detection tests.

### Virion Particle Morphology and Cytopathology of the Infected Plant Tissues

The pretreatment of plant tissues for TEM analyses was carried out as previously described (Ramos-González et al., 2017). Copper grids with plant tissue sections embedded in Spurr’s epoxy resin and stained with 3% uranyl acetate and Reynold’s lead citrate were examined in a JEOL JEM 1011 (JEOL, Akishima, Japan) transmission electron microscope. For particle size measurements, all images were recorded at the same magnification (200,000x). Ten to fifteen tissue sections per virus/host pair from four independent plants were examined. Exceptionally, virions of the sample Cdb1 corresponded to a single plant. At least 50 particles per virus/host combination were measured using the program ImageJ (Collins, 2007). The measurements of length and width were carried out from different particles. For the sake of comparative analysis of the virion morphology, viral particles in the samples SPa1 and Prb1 were measured in tissues from both the naturally infected *Ligustrum* spp. and *Arabidopsis thaliana* plants which were experimentally infected using viruliferous mites found in field-collected branches from those samples. Virions of the sample Cdb1 were only measured in tissues of an *L. sinense* plant. In parallel, particles of the typical cilevirus PfGSV isolate BSB infecting both arabidopsis and *L. japonicum* plants were measured. Values of viral width and length were compared using the non-parametric tests Kruskal-Wallis and Wilcoxon signed-rank implemented in Rstudio (Rstudio_Team, 2021). Row data were represented using a violin plot using geom_violin (ggplot2, tidyverse) also implemented in Rstudio.

### RNA Extraction, cDNA Synthesis, and Virus Detection

One to two grams of plant tissues were ground in mortars in presence of liquid N_2_ and their RNA extracts were obtained using TRIzol™ reagent following the recommendation of the manufacturer (Life Technologies, Foster City, CA, United States). No more than 100 mg of plant tissue were processed with 1 mL of TRIzol™. The RNA concentration in the final extracts was determined at 260 nm using a Nanodrop ND-8000 micro-spectrophotometer (Thermo Scientific, Waltham, MA, United States). Approximately 500 ng of the RNA extracts were used to prepare the cDNA solutions using a mix of random hexamer primers and GoScript™ Reverse Transcriptase kit as described by the manufacturer (Promega, Madison, WI, United States).

For the preparation of RNA extracts from small pieces of tissues, i.e., isolated leaf lesions comprising no more than 10–30 mg, tissue fragments were ground using the FastPrep-24™ homogenizer (MP Biomedicals, Santa Ana, CA, United States). Samples were ground in the presence of three steel beads of 3 mm in diameter and 500 μL of TRIzol™ during two cycles of 30 s at 6.5 m s^–1^. After the purification of the total RNA fraction, pellets were resuspended in 10 μL of Milli-Q water treated with DEPC. cDNA solutions were prepared using 2 μL of the RNA extracts and following the directions described in the GoScript™ Reverse Transcriptase kit (Promega).

The presence of viral genomes was tested by PCR using 2 μL of cDNA solutions as templates and GoTaq^®^ G2 Green Master Mix (Promega). A set of specific and generic primers were used for the detection of the cileviruses CiLV-C, CiLV-C2, PfGSV, and the partially characterized kitavirus SvRSV (Table 1). Newly designed primer pairs obtained after genomic analyses carried out in this study were also used for viral screening (Table 1). Generated amplicons were separated and visualized in 1% agarose gel in 1X Tris-acetate-ethylenediaminetetraacetic acid (TAE) in the presence of ethidium bromide (0.5 μg/mL), under ultraviolet light.

**TABLE 1:** List of specific and generic primers for the detection of typical cileviruses and cile-like viruses described in this study.

Virus^a^	ORF target	Primer sequence (5′-3′)	Ta^b^ (°C)	Amplicon size (bp)	References
CiLV-C	*p24*	F: CGCAGTTTCCTAATAACACC	54	322	Chabi-Jesus et al., 2021
		R: GGGAGTTCAGCATAAAGC			
CiLV-C CRD	*p29*	F: CAGAAGGCCGAGGTTGTAAAG	56	330	Ramos-González et al., 2016
		R: GTAGTGATCACTGAACTCGAATACC			
	*p24*	F: ATGTTGGCAACGGAAAGT	54	522	Chabi-Jesus et al., 2021
		R: AACTTTTTCAACCCTGTTCAC			
CiLV-C SJP	*p29*	F: GTAARCAAAAGGTCGAGGTTGTCC	56	456	Ramos-González et al., 2016
		R: TCTGTTGTCTAGCAGCRAGTAATG			
	*p24*	F: CTCATGATATCCTTGATGACC	54	393	Chabi-Jesus et al., 2021
		R: CAACCTTCTCAACCTTATTAGTC			
CiLV-C2	*p29*	F: ATGAGTAACATTGTGTCGTTTTCGTTGT	56	795	Roy et al., 2013
		R: TCACTCTTCCTGTTCATCAACCTGTT			
PfGSV	*p29*	F: ACACCAAGAGTACTATCGATC	54	452	Ramos-González et al., 2020
		R: CATCAAGTGGAGCAAGTTC			
SvRSV	*RdRp*	F: TGTCGAACTTTGGTATGAGTCG	54	596	Ferreira et al., 2007
		R: CCGGTTCGTCAAATAACTCC			
	*p31*	F: CACGTCGTTCAGCAGAA	54	490	This study
		R: ACCTCTTGGTCATCGACT			
	*p23*	F: GGCTGTTCTGGTATTATTTG	54	474	
		R: GAACTCAAGGTACTAGAAG			
LigCSV	*p31*	F: TGGTTACCGTTACTTTTTCTC	54	123	
		R: TTTTAGACTTCAACGCCTTC			
		F: GGTCACGAATTATAAGGCAG	54	373	
		R: TGGAATGGCTTTGATAGTCT			
	*p23*	F: TCGGATTGATTGTCTCTGTG	54	420	
		R: AAACCGGATTTGAATTATATG			
		F: CTCTCATAAATTGGGCGAAG	54	304	
		R: GGCAACTCCTTGTAAAGTTT			
	*p33*	F: GGATATACTCTCGAGCGATT	54	318	
		R: CCCATTTCAGAACCAACATT			
LigLV	*RdRp*	F: AAAACCCACACTTTCTGATG	54	303	
		R: TTGCACTCGAATAACAAGAC			
	*p32*	F: AAATCAGGCTGTTAATGTCG	54	435	
		R: AGGACACGCAAATTCTTATG			
	*p34*	F: TTGTCTCTAATGGATCCGAG	54	391	
		R: GCATTTTCATTTACGCTGTC			
	*p24*	F: CATGTATGTAGCAGTGTTGG	54	316	
		R: GAGAATTCGCGTTATTGGAT			

*^a^CiLV-C, citrus leprosis virus C; CiLV-C CRD, citrus leprosis virus C strain CRD; CiLV-C SJP, citrus leprosis virus C strain SJP; CiLV-C2, citrus leprosis virus C2; PfGSV, passion fruit green spot virus; SvRSV, Solanum violifolium ringspot virus; LigCSV, Ligustrum chlorotic spot virus; LigLV, Ligustrum leprosis virus.*

*^b^Ta: PCR annealing temperature. F and R indicate forward and reverse primers, respectively.*

### High Throughput Sequencing and *in silico* Assembly of Viral Genomes

RNA extracts obtained by TRIzol™ method from the samples Prb1, Cdb1, SPa1, and Crb1 were further purified using RNeasy Mini Kit (QIAGEN, Venlo, The Netherlands). The concentration of RNA in the final solutions and the A260/A280 ratio were assessed using a NanoDrop ND-8000 micro-spectrophotometer. One to five hundred nanograms of each RNA extract were sent to the Animal Biotech Laboratory at Escola Superior de Agricultura Luiz de Queiroz, University of São Paulo (Piracicaba, SP, Brazil) for high-throughput sequencing (HTS) using HiSeq 2500 Technology (2 × 150 nt paired-end reads) (Illumina, San Diego, CA, United States). RNA extracts from each sample were independently processed resulting in four HTS libraries. The enrichment of poly-A RNA fractions, preparation of libraries, sequencing, quality assessments of reads, and the removal of the adaptor sequences, were carried out as previously described (Chabi-Jesus et al., 2021). Reads were *de novo* assembled using SPAdes (Bankevich et al., 2012) and Trinity (Haas et al., 2013) implemented on the Galaxy platform^1^ v 21.09 (Afgan et al., 2018). Contigs were annotated with BlastX and BlastN implemented in Geneious software package v 11.1.4 (Kearse et al., 2012) using custom-organized plant viral genome databases retrieved from NCBI Virus^2^ (Hatcher et al., 2017). The largest contigs producing the best *E-value* score with cileviruses sequences i.e., CiLV-C, CiLV-C2, and PfGSV, were selected for further detailed analyses. Nucleotide sequences corresponding to the genomic fragments of the partially characterized SvRSV isolates previously collected in Brazil and Argentina, i.e., GenBank # DQ514336 and KT733671, were also included in the nucleotide database during the Blast-based screenings of the contigs.

Three sets of primers corresponding to each of the kitaviruses identified in this study were designed using the Primer3 program (Untergasser et al., 2012). Primer target regions were conveniently selected to generate overlapping amplicons of approximately 0.7–1.0 kb (Supplementary Table 1). Generated amplicons were used for the validation of HTS results and, when required, to amplify the genome sequence of novel viral isolates. The 5′-ends of genomic molecules of new viruses were obtained using SMARTer^®^ RACE 5′/3′ Kit (Clontech Laboratories, Mountain View, CA, United States) following the procedure previously described (Ramos-González et al., 2020).

The relative abundance of viral-specific reads and mean coverage of viral bases per generated HTS library were calculated using BBMap (Bushnell, 2014). Reads were mapped to each genomic segment of the virus detected in each sample.

### *In silico* Viral Genome Analyses: Annotation, Pairwise Comparisons, and Phylogenetic Relationships

Viral ORFs were identified using the ORF finder.^3^ The presence of signal peptides, conserved domain architecture, and transmembrane helices in predicted viral proteins was detected using SignalP 5.0^4^ (Armenteros et al., 2019), MOTIF Search,^5^ TMHMM Server 2.06^6^ (Sonnhammer et al., 1998), and Deeploc v 1^7^ (Armenteros et al., 2017), respectively. Nucleotide and predicted amino acid sequences were aligned and the identity values were assessed using MAFTT^8^ (Katoh and Standley, 2013).

For phylogenetic analyses, sequences from kitaviruses, unclassified kita/nege like viruses including those of the groups negevirus, centivirus, and aphiglyvirus, were retrieved from GenBank after a BLAST search (cut-off *E-value* < e-10) using typical cilevirus sequences as the query. Some viruses of the family *Virgaviridae* were also included as an outgroup. Phylogenetic informative regions of the multiple sequence alignments (MSAs) were selected using BMGE software (Criscuolo and Gribaldo, 2010) implemented in NGPhylogeny^9^ (Lemoine et al., 2019). BMGE parameters were set as follows: estimated matrix BLOSUM 62, sliding windows size = 7, maximum entropy threshold = 0.5, gap rate cut-off = 0.5, and minimum block size = 0.5. The substitution models with the lower Bayesian information criterion scores for RdRp and P24 MSAs and the Maximum Likelihood trees were obtained using W-IQ-TREE software v. 1.6.12^10^ (Trifinopoulos et al., 2016). The reliability of the inferred evolutionary relationships was assessed by 1,000 bootstrap replications. Sequences of the methyltransferase and helicase domains, and RdRp encoded by the RNA1 and RNA2 molecules, respectively, of the blunerviruses blueberry necrotic ring blotch virus of the strains Georgia and RL (BNRBV_Georgia and BNRBV_RL, respectively), TPNRBV, and tomato fruit blotch virus (ToFBV) were concatenated as previously described (Quito-Avila et al., 2013; Ramos-González et al., 2020). Trees were edited and visualized using Interactive Tree Of Life (iTOL) v 5 (Letunic and Bork, 2021).

### Identification of Conserved 3′Untranslated Regions and *in silico* Search for Novel Viral Segments

Viral segments of SvRSV, LigCSV, LigLV, and those of typical cileviruses were aligned using MAFTT (Katoh and Standley, 2013) and visualized using the Jalview program (Procter et al., 2021). Sequences comprising 90–120 nts of the highly conserved 3′ untranslated regions (UTR) of SvRSV, LigCSV, and LigLV were used to search for the presence of putative non-detected viral segments in the HTS-contigs libraries using the UTR-backed iterative BLASTN approach (Zhang et al., 2022). Previously, BLASTN databases were built from the HTS-contig libraries of each sample using Geneious software package v 11.1.4 (Kearse et al., 2012).

### Experimental Viral Transmission

*Brevipalpus* mites collected from branches of the privet sample SPa1 were directly transferred to healthy *Arabidopsis thaliana* Col-0 plants. Ten to fifteen mites were distributed onto each plant at a maximum of five mites per leaf. Seven to ten days after the transfer, mites were collected and kept in 90% ethanol solution while waiting for further morphological characterization. Arabidopsis leaves were collected when chlorotic and yellowish blotches were visually detected. Fragments of the symptomatic areas were conserved in Karnowski solution for TEM analyses or processed for RNA extraction and viral detection. Arabidopsis plants were kept at 23 ± 1°C and 14 h of light in a controlled growth chamber (Adaptis AR A1000, Conviron, Winnipeg, MB, Canada) from their germination to the end of the experiments. Two independent experiments were conducted in two seasons in which groups of four and seven arabidopsis plants were used for mite infestations.

### Morphological Identification of *Brevipalpus* Mites

Mites prefixed in 90% ethanol solution were mounted in Hoyer’s medium before the examination by differential interference contrast in a Zeis Axioimager II microscope (Carl Zeis AG., Jena, Germany). When required, other specimens were processed for observation under a JEM IT 300 scanning electron microscope (JEOL) following protocols previously described (Ramos-González et al., 2017). Distinctive external structures of mites, i.e., number of dorsolateral setae, number of solenidia on the tarsus of leg II, dorsal and venter reticulations patterns, the shape of vesicle of the spermatheca, and morphology of microplates were visually evaluated under microscopy and compared with the holotypes considering the detailed descriptions available (Beard et al., 2015). All images were digitally recorded.

## Results

### Detection of Solanum Violifolium Ringspot Virus and Cilevirus-Like Particles in Symptomatic Samples

Plant samples collected in this study were selected based on their infection by partially characterized or unknown viruses likely to be members of the family *Kitaviridae* according to previous reports (Vergani, 1942; Lima et al., 1991; Lima Neto et al., 1994; Ferreira et al., 2007; Kitajima et al., 2010). Regardless of the plant species, these samples showed chlorotic or yellowish blotches in leaves (Figure 1). Nearly concentric rings of necrotic tissues were frequently observed in *S. violifolium* infected leaves likely in advanced stages of disease progression (Figure 1A). Small necrotic points were also observed in the central area of lesions of privet leaves of the samples Crb1 and Crb2 (Figures 1C,D). In leaves of *S. violifolium* and *Ligustrum* spp. plants, regular chlorotic concentric rings coexisted with asymmetrical chlorotic spots. Generally, symptomatic leaves were unevenly distributed across the plants suggesting non-systemic diseases.

RNA extracts from all samples were tested by RT-PCR using specific primers for the detection of the cileviruses CiLV-C, CiLV-C2, PfGSV, and the partially characterized SvRSV. Bands of approximately 600 bp were obtained in samples Prb1, Crb1, and Crb2 from the reactions using SvRSV-specific primers (Figure 1F). Reactions using primers for the detection of typical cileviruses were negative (results not shown).

**FIGURE 2:**
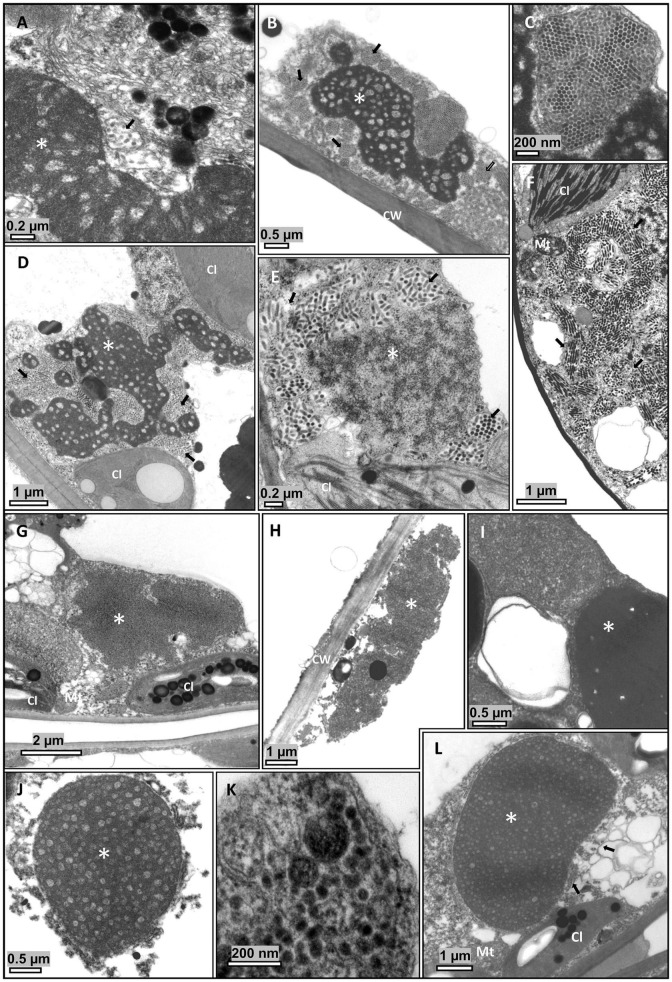
Transmission electron micrographs of ultrathin sections of symptomatic tissues from leaves of *Ligustrum* spp. plants. Short, bacilliform, membrane-bounded particles, presumed virions, are usually observed close to the viroplasm, contained in membrane-bound cisternae of the endoplasmic reticulum. **(A–C)** *Ligustrum lucidum*, sample Crb1. **(D–F)** *L. japonicum*, sample Crb2. **(G–I)** *L. sinense*, sample Cdb1. **(J–L)** *L. japonicum*, sample SPa1. **(A–L)** Electron dense, vacuolated masses of varying shapes and bacilliform particles are observed in the cytoplasm of cells of the epidermis, mesophyll parenchyma, and, occasionally, in the xylem parenchyma. **(G–H)** Non-vacuolated viroplasms. Cl, chloroplast; CW, Cell wall; Mt, mitochondrion; V, virion. *Viroplasm.

In parallel analyses using TEM, presumed virus particles were observed in all symptomatic samples (Figure 2). Characteristic cytopathologic features of cilevirus infections, including the presence of viroplasm, were distinguished (Figures 2A,B,D,E,I,J,L). Viroplasms are electron-dense vacuolated structures of wide-ranging sizes and shapes that are believed to be formed by aggregates of virus-encoded proteins. Non-vacuolated viroplasms were observed in some samples (Figures 2G,H). In a large proportion of cells of those samples, viroplasms could not be detected despite the high number of presumed viral particles. Short, bacilliform or spheroidal, membrane-bounded particles, likely virions, were observed inside cisternae of the endoplasmic reticulum (Figures 2C,F,K). Viral particles were also observed in the cytosol of *S. violifolium* plants (pictures not shown). Cytopathic effects in *S. violifolium* cells were similar to those previously observed (Ferreira et al., 2007; Arena et al., 2017).

### Plant Virome Analyses Reveal the Complete Genomes of Solanum Violifolium Ringspot Virus and Two Other Related but Distinct Types of Cile-Like Viruses

The composition of the RNA extracts from the samples Crb1, SPa1, Cdb1, and Prb1 was investigated through HTS and independent libraries with more than 12 million reads were obtained (Table 2). After *de novo* assembly of reads, generated contigs were examined using BlastN and BlastX for global detection of viral sequences, and in particular, of kita-like viruses. Contigs of approximately 8 kb with a high-quality match (*E-value* ≈ 0) to the previously described genomic fragments of SvRSV (597 nts of the ORF *RdRp*, RNA1, GenBank accession numbers DQ514336 and KT733671) were detected in samples Crb1 and Prb1. In these samples, contigs of ≈ 3 kb in length were also detected. They showed the best matches (*E*-value ≈ 10^–30^) with the RNA2 segments of cileviruses. The sample Crb1 was particularly relevant since another pair of contigs, also comprising kitavirus-like sequences, were identified in its library. The largest contig comprised cilevirus RNA2-like sequences, whereas the smallest one, with approximately 1.0 Kb, contained sequences with certain nt sequence identity with the ORF *p29* of cileviruses. According to the low nt sequence identity detected, the two pairs of contigs in the sample Crb1 likely belonged to viruses with markedly different genomic sequences. Interestingly, the second pair of contigs identified in the sample Crb1 displayed the best matches with the kitavirus-related contigs of approximately 8 and 3.5 kb that were independently recovered from libraries of the samples SPa1 and Cdb1. In summary, bioinformatics analyses revealed the near-complete genome sequences of two isolates of the partially characterized kitavirus SvRSV and three unknown kitaviruses.

**TABLE 2:** Description of high-throughput sequencing libraries obtained in this study.

Field sample	Crb1				Prb1		SPa1		Cdb1	
Total number of reads recovered by library	13,181,765				16,000,302		15,055,185		12,574,635	
Identified virus_isolate	SvRSV_Crb1^a^		LigCSV_Crb1		SvRSV_Prb1		LigCSV_SPa1		LigLV_Cdb1	
Viral segment	RNA1	RNA2	RNA1	RNA2	RNA1	RNA2	RNA1	RNA2	RNA1	RNA2
Length of viral genomic segments (nts)	8,635	3,641	8,373	3,612	8,658	3,622	8,383	3,681	8,893	3,743
Reads mapped to viral genome	89,906	59,636	1,058	6,260	5,378,852	774,604	42,519	134,316	229,140	214,082
Viral-derived reads in the library (%)	0.682	0.452	0.008	0.047	33.618	4.841	0.282	0.892	1.822	1.702

*^a^GenBank accession numbers of each molecule are the following: SvRSV_Prb1 (OK626439 and OK626440), SvRSV_Crb1 (OK626441 and OK626442), LigCSV_SPa1 (OK626447 and OK626448), LigCSV_Crb1 (OK626449 and OK626450), and LigLV_Cdb1 (OK626451 and OK626452).*

Kitavirus-related contigs were used to generate specific primers (Supplementary Table 1) whereby the cDNA extracts from each plant sample were tested. Sanger-obtained sequences of the amplicons were aligned with the sequence of contigs recovered from the HTS method. Successive heuristic approaches based on the testing all the studied plant samples with the specific primer sets and nucleotide sequence comparisons of the generated amplicons revealed the full picture of the kitavirid diversity in the samples as follows: isolates of SvRSV were detected in samples Prb1 and Crb1, whereas isolates of a tentative novel virus called Ligustrum chlorotic spot virus (LigCSV) were identified in samples SPa1 and Crb1, and an isolate of a second tentative novel virus called Ligustrum leprosis virus (LigLV) was identified in the sample Cdb1. 5′- RACE analyses were conducted for the isolates SvRSV_Prb1, LigCSV_SPa1, and LigLV_Cdb1. The near-complete sequence of LigCSV isolate Crb1 was assembled from a mix of HTS- and Sanger-obtained contigs.

**FIGURE 3:**
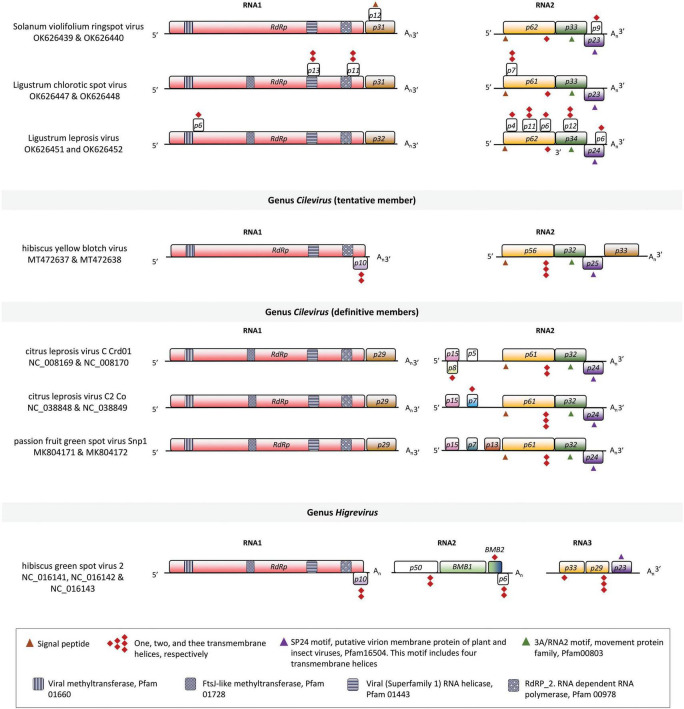
Linear genomic maps of Solanum violifolium ringspot virus, Ligustrum chlorotic spot virus, Ligustrum leprosis virus, and other tentative and definitive members of the family *Kitaviridae*. Open reading frames (ORFs) are represented by boxes. Colors indicate a putative conserved functional or structural relationship between ORFs from different viruses. In citrus leprosis virus C, genus *Cilevirus, RdRp*: RNA dependent-RNA polymerase, *p29*: putative coat protein, *p15*: putative RNA silencing suppressor, *p61*: putative glycoprotein, likely a structural protein of the virion, *p32*: movement protein, *p24*: putative structural protein of the virion. White boxes indicate unknown features. Symbols of black pattern-filled boxes, triangles, and diamonds depict relevant amino acid motifs as described in the legend box.

Excluding the polyA tails detected in the sequences, the genome of SvRSV_Prb1 comprises 12,232 nts split into two segments identified as RNA1 of 8,622 nts and RNA2 of 3,610 nts (GenBank accession numbers OK626439 and OK626440, respectively) (Figure 3). Similarly, the genome of LigCSV_SPa1 has 12,022 nts distributed in the segments RNA1 of 8,369 nts and RNA2 of 3,653 nts (GenBank acc. num. OK626447 and OK626448, respectively), whereas the genome of LigLV_Cdb1, with 12,611 nts in sum, contains 8,879 nts in the RNA1 and 3,732 in its RNA2 (GenBank acc. num. OK626451 and OK626452, respectively). Without RACE analyses, the near-complete genomes of SvRSV of the isolate Crb1 (GenBank # OK626441 and OK626442) and LigCSV_Crb1 (OK626449 and OK626450) were found to be slightly shorter than those of the isolate Prb1 and SPa1, respectively. Nucleotide sequences from different isolates of the same virus showed very high identity values (>93%), i.e., among the two isolates of SvRSV, and the two isolates of LigCSV (Table 3). Percent identities of the comparisons of the genomic sequences from different viruses ranged from 49.83 to 69.07%. Higher values corresponded to the comparisons involving the RNA1 molecules of LigCSV and LigLV (68–69%). Overall, the RNA2 molecules were more divergent than the RNA1 molecules.

**TABLE 3:** Nucleotide sequence identity amongst the RNA segments of viruses described in this study.

RNA1/RNA2	LigLV_Cdb1	LigCSV_Crb1	LigCSV_SPa1	SvRSV_Crb1	SvRSV_Prb1
SvRSV_Prb1^a^	52.91	52.52	52.60	**98.12**	
SvRSV_Crb1	52.63	52.59	52.65		**98.65**
LigCSV_SPa1	69.07	**98.87**		50.66	50.55
LigCSV_Crb1	68.97		**93.96**	50.66	50.61
LigLV_Cdb1		61.63	62.25	49.83	49.90

*Figures above and below the diagonal depict the RNA1 and RNA2 identity values, respectively. The identity values higher than 90% are highlighted in boldface. ^a^GenBank accession numbers of each molecule are the following: SvRSV_Prb1 (OK626439 and OK626440), SvRSV_Crb1 (OK626441 and OK626442), LigCSV_SPa1 (OK626447 and OK626448), LigCSV_Crb1 (OK626449 and OK626450), and LigLV_Cdb1 (OK626451 and OK626452).*

Since three clusters of viral sequences could be consistently observed upon the analysis of genomic comparisons, one isolate of each virus was chosen for further *in silico* studies. The genomes of SvRSV_Prb1, LigCSV_SPa1, and LigLV_Cdb1 contain five ORFs larger than 500 nts distributed two in the RNA1 segment and three in the RNA2 (Figure 3). The RNA1 molecules of SvRSV, LigCSV, and LigLV have a large ORF (>7,200 nts) that according to BlastX encode the RdRp protein and a second ORF, likely an ortholog of the *p29* in typical cileviruses. The RdRp of these viruses had the motifs RdRP_2 (PF00978), Viral_helicase1 (PF01443), V_methyltransferase (PF01660), and AAA_30 (PF13604) in common, whereas other motifs were exclusively found in either the RdRp of SvRSV or LigLV, e.g., Chropara_Vmeth (PF19223, motif present in a family of chroparavirus proteins which are likely methyltransferases involved in mRNA capping), and DUF5488 (PF17590, motif in proteins of unknown function found in orthopoxvirus) (Supplementary Table 2).

RNA2 molecules in SvRSV_Prb1, LigCSV_SPa1, and LigLV_Cdb1 comprise three major ORFs encoding proteins possessing features similar to those in cileviruses (Figure 3). The larger protein encoded in RNA2 is a putative glycoprotein. *In silico* analyses revealed the presence of a signal peptide that in the case of P62 from SvRSV_Prb1 has a predicted cleavage site between the aa positions 17 and 18: VL**K**|**F**E. In P61 and P62 of LigCSV_SPa1 and P62 of LigLV_Cdb1, the signal peptides are larger with cleavage sites between positions 24 and 25: VN**A**|**T**I, and 20 and 21: TL**S**|**Y**V, respectively. These three proteins have theoretical N-glycosylation sites predicted in position **N**_142_ASR in SvRSV_Prb1, **N**_152_HSE, **N**_175_LTR, and **N**_235_FTN in LigCSV_SPa1, and N_91_VST, **N**_152_HSE, **N**_175_LTR, and **N**_235_FTN in LigLV_Cdb1. Invariably, these putative glycoproteins from the three viruses also show transmembrane domains near their COOH ends. Immediately downstream of the ORFs *p61-62*, the ORFs *p33-34* encode proteins with the characteristic 3A motif (PF00803) of viral movement proteins. The 3′-end of ORFs *p33-34* partially overlaps the 5′-end of the ORFs *p23-24*. The proteins encoded by ORF *p23-24* harbor the motifs SP24 (PF16504) which is centrally placed in the protein and at the structural level possesses four transmembrane helixes.

Other fully or partially overlapping ORFs smaller than 500 nts were detected over the genome of the studied viruses (Figure 3). In the three viruses, *in silico* analyses of the putative structure of the predicted encoded polypeptides indicated the presence of up to two transmembrane helices per molecule (Supplementary Table 3). Predicted small transmembrane (TM) proteins were more frequently detected in the RNA2 of LigLV, i.e., three with one TM helices, and two showing two TM helices. A putative basic protein (pI = 12.9) of approximately 12 kDa encoded in the RNA1 of SvRSV_Prb1 (ORF of 315 nts, positions 7,605–7,919) shows a signal peptide, which according to *in silico* analysis using DeepLoc, might suggest its interaction with the secretory pathway machinery. None of these predicted proteins had a significant identity with any protein described in public databases.

Paired comparisons among ORFs and their deduced proteins of SvRSV, LigCSV, and LigLV denoted the highest identity values between genes from LigCSV and LigLV (Table 4). Maximum identity percentages for each genomic segment corresponded with ORFs *RdRp* (nts: 70.5; aa: 74.7) and *p23-24* (nts: 75.7; aa: 83.1), in the RNA1 and 2, respectively. The highest values of identity in the evaluations including SvRSV were concentrated on the same ORFs, but they were consistently lower. For instance, amino acid sequence identities in the comparisons of RdRp and P23 from SvRSV with LigCSV and LigLV reached no more than 45 and 57%, respectively. When these analyses were expanded to definitive and tentative kitaviruses of the genera *Cilevirus* and *Higrevirus*, the higher identity values were invariably observed in the comparisons involving genes from the typical cileviruses, i.e., CiLV-C, CiLV-C2, and PfGSV (Table 4). In comparisons where orthologous genes from the higrevirus hibiscus green spot virus 2 (HGSV2) were included, the obtained values were almost as low as those obtained in the comparison concerning ORFs from the cile-like virus HYBV.

**TABLE 4:** Nucleotide (nt) and deduced amino acid (aa) identities between SvRSV_Prb1, LigCSV_SPa1 and LigLV_Cdb1, and other tentative and definitive members of the family *Kitaviridae*.

Virus	Genome segment	ORF (nts)^a^	Theoretical pI/MW (kDa)^b^	Cile-like viruses				Typical cileviruses			Higrevirus
				LigLV_Cdb1	LigCSV_SPa1	HYBV	PisVY	CiLV-C_Crd1	CiLV-C2_Co	PfGSV_Snp1	HGSV2
				
				Nucleotide/deduced amino acid sequence identity (%)							
SvRSV_Prb1^c^	RNA1	*RdRp* (7,419)	6.25/283.14	52.1/**44.5**	51.8/**44.2**	47.6/37.5	50.3/40.2	51.7/43.5	51.8/**44.7**	51.3/**44.3**	45.8/32.3
		*p31* (849)	8.64/30.65	41.6/27.8	43.4/28.6	38.1/21.2	37.5/21.0	42.7/27.5	39.6/27.9	41.7/**31.0**	*^d^
	RNA2	*p62* (1,665)	7.13/62.15	41.8/25.4	43.1/**26.2**	38.5/17.8	35.4/18.5	42.1/24.0	42.0/24.1	41.1/24.4	*
		*p33* (900)	7.81/32.97	51.7/44.4	51.8/43.0	50.7/43.4	54.0/44.7	57.5/**48.6**	57.4/**50.2**	54.7/**49.8**	*
		*p23* (633)	9.56/23.43	58.8/**55.1**	58.2/**56.6**	47.7/37.1	55.8/49.7	58.7/52.1	58.3/52.4	59.2/**53.4**	42.7/25.1
LigCSV_SPa1	RNA1	*RdRp* (7,230)	7.41/276.11	70.5/**74.7**		49.7/37.0	50.3/39.4	58.0/54.7	57.0/53.2	58.4/54.4	44.4/32.2
		*p31* (849)	9.02/30.63	57.4/**47.5**		38.4/20.8	38.6/22.7	46.8/33.2	44.3/34.1	45.6/31.1	*
	RNA2	*p61* (1,596)	5.81/60.56	58.4/**50.1**		41.3/19.6	39.3/19.6	43.9/28.5	43.8/28.7	42.6/29.9	*
		*p33* (915)	7.67/33.34	63.3/**59.9**		51.4/42.6	54.8/40.8	56.2/53.9	57.2/50.5	56.2/53.0	*
		*p23* (624)	9.36/23.23	75.7/**83.1**		47.9/39.6	56.5/51.3	64.2/68.3	63.6/65.4	64.8/66.0	46.2/31.4
LigLV_Cdb1	RNA1	*RdRp* (7,578)	6.80/288.80		70.5/**74.7**	48.9/36.2	51.0/40.0	57.8/53.7	57.4/52.0	57.7/52.3	45.0/31.5
		*p32* (885)	9.05/31.98		57.4/**47.5**	38.3/21.9	36.4/20.5	45.0/29.8	43.7/31.4	41.0/29.0	*
	RNA2	*p62* (1,605)	6.33/61.55		58.4/**50.1**	38.8/17.6	40.3/17.9	44.5/31.0	45.7/33.3	45.8/31.7	*
		*p34* (924)	7.64/33.83		63.3/**59.9**	50.3/42.4	52.5/41.3	56.6/54.3	57.3/53.1	55.1/52.1	*
		*p24* (630)	9.47/23.57		75.7/**83.1**	47.7/37.19	58.1/51.2	62.7/66.5	63.7/62.9	65.3/63.6	45.0/27.5

*The highest values of nt and aa identity in each row are underlined and highlighted in bold. ^a^Length (nts) of each ORF including the stop codon. ^b^The isoelectric point (pI) and molecular weight (MW) in kDa of the deduced polypeptides were assessed using the Compute pI/Mw tool available in https://web.expasy.org/compute_pi/. ^c^GenBank or RefSeq accession numbers of each molecule are the following: SvRSV_Prb1 (OK626439 and OK626440), SvRSV_Crb1 (OK626441 and OK626442), LigCSV_SPa1 (OK626447 and OK626448), LigCSV_Crb1 (OK626449 and OK626450), and LigLV_Cdb1 (OK626451 and OK626452), CiLV-C Crd1 (NC008169 and NC008170), CiLV-C2_Co (NC038848 and NC038849), PfGSV_Snp1 (MK804171 and MK804172), HYBV (MT472637 and MT472638), PisVY (MT362606 and MT362605), and HGSV2 (NC_016141, NC_016142, and NC_016143). ^d^Absence of orthologues genes.*

**FIGURE 4:**
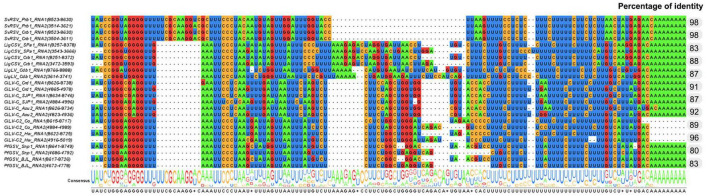
Identification of conserved nucleotide sequences in the 3′-end genomic segments of isolates of Solanum violifolium ringspot virus (SvRSV), Ligustrum chlorotic spot virus (LigCSV), and Ligustrum leprosis virus (LigLV). Values in the column on the right indicate the nucleotide sequence identity between the 3′-termini of RNA1 and RNA2 viral segments. Genomic sequences of several isolates of the cileviruses CiLV-C, CiLV-C2, and PfGSV were also included in the study highlighting conserved nucleotide stretches across definitive and putative members of the genus *Cilevirus*.

The alignment of RNA1 and RNA2 of SvRSV, LigCSV, and LigLV revealed the presence of conserved nucleotide stretches of ≈90–120 nts between the 3′-termini of each segment (Figure 4). In SvRSV isolates, these regions were more similar (98% nt sequence identity) than in LigLV and LigCSV (83–88% nt sequence identity). When the 3′-termini of the cileviruses CiLV-C, CiLV-C2, and PfGSV were included in the alignment, the presence of highly conserved nucleotide sequences in all these viruses became evident, likely denoting the putative functional role of these sequences as described in other viruses (Dreher, 1999; Liu et al., 2020; Rasekhian et al., 2021). The implementation of the UTR-backed iterative BLASTN approach (Zhang et al., 2022) using the 3′-terminus conserved nucleotide sequences of SvRSV, LigCSV, and LigLV, resulted in the recovery of contigs corresponding to the RNA1 and RNA2 segments already known, and new putative genomic segments were not identified.

**FIGURE 5:**
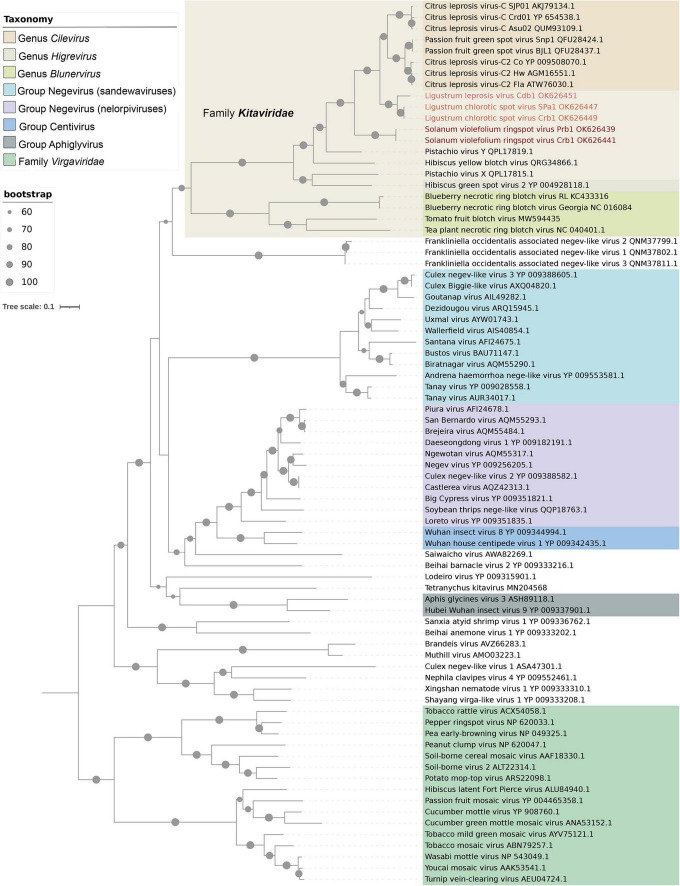
Phylogenetic reconstruction for viruses of the family *Kitaviridae*. Isolates of Solanum violifolium ringspot virus, Ligustrum chlorotic spot virus, and Ligustrum leprosis virus are highlighted in different red tones. The maximum-likelihood phylogenetic tree is based on the deduced amino acid sequences of the RNA-dependent RNA polymerase. The tree was rooted using viruses of the family *Virgaviridae* as an external group. Phylogenetic informative regions of the multiple sequence alignment included 546 residues that were selected using BMGE software (Criscuolo and Gribaldo, 2010) and its evolutionary history was inferred based on the model LG+F+I+G4 (Le and Gascuel, 2008). The bootstrap support values (1,000 replications) of branches greater than 60% are indicated with solid black circles next to the corresponding nodes. The scale bar specifies the average number of amino acid substitutions per site.

A maximum-likelihood tree based on the deduced aa sequences of RpRp clustered SvRSV, LigCSV, and LigLV into two branches (Figure 5). They were placed in an intermediary position between branches comprising typical cileviruses, the cile-like virus HYBV, and the partially characterized cile-like virus Pistachio virus Y (GenBank accession numbers MT362606 and MT362605). Isolates of LigCSV and LigLV were grouped closer to typical cileviruses, whereas isolates of SvRSV were grouped in a more basal position. Overall, kitaviruses from the three genera were monophyletically clustered in a highly bootstrap-supported branch (>80%). Members of the family *Kitaviridae* shared a branch with three viruses found associated with the thrips *Frankliniella occidentalis* in Italy (Chiapello et al., 2021). *Frankliniella occidentalis* associated negev-like virus 1 (Foanegev1), Foanegev2, and Foanegev3 may belong to a new clade of arthropod-infecting viruses that, although related to negeviruses, have a distinct genomic organization. Subsequently, kitaviruses and *Frankliniella occidentalis* associated viruses clustered in a larger branch which also includes nelorpiviruses and sandewaviruses of the proposed taxon Negevirus, the centiviruses Wuhan insect virus 8, and Wuhan house centipede virus 1, and several other kita-like viruses, e.g., Saiwaicho virus and Beihai barnacle virus 2 (Kondo et al., 2020). This larger branch was not supported by bootstrap values > 60%.

In the tree using the P24 protein, SvRSV, LigCSV, and LigLV had almost the same phylogenetic pattern observed in the RdRp tree (Supplementary Figure 1). They were subclustered in a branch comprising the typical cileviruses and, in more basal positions, the cile-like virus HYBV, and the higrevirus HGSV2. In contrast to the RdRp-based tree, blunerviruses grouped in a sister branch that also included nelorpiviruses, centiviruses, aphiglyviruses, and other nege-kita/related arthropod-infecting viruses, e.g., Tetranychus urticae kitavirus.

### *Arabidopsis thaliana* Plants Are Susceptible to Infection by Ligustrum Chlorotic Spot Virus

**FIGURE 6:**
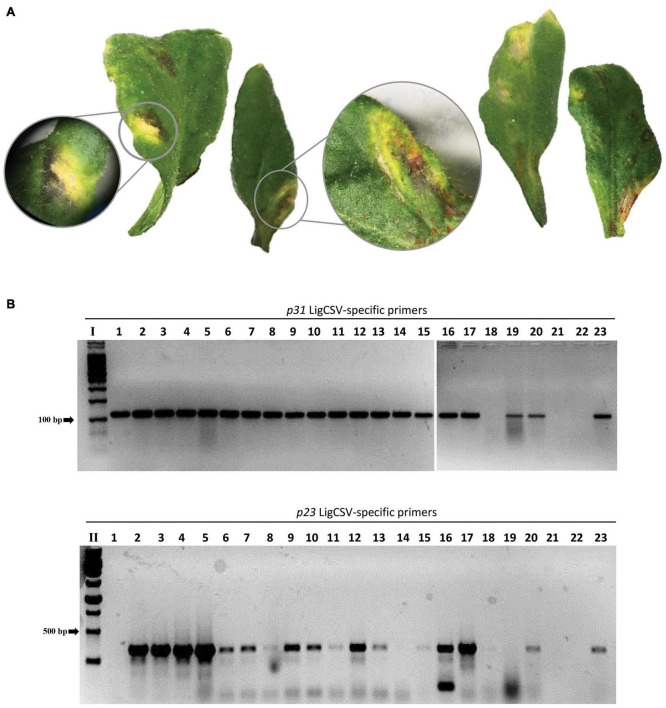
Experimental transmission of Ligustrum chlorotic spot virus (LigCSV) to healthy *Arabidopsis thaliana* plants using viruliferous *Brevipalpus papayensis* mites. **(A)** Arabidopsis leaves showing chlorotic and necrotic lesions after 7 days of infestation with *Brevipalpus papayensis* found in symptomatic *Ligustrum japonicum* plants infected with LigCSV, collected in São Paulo, SP, Brazil. **(B)** 1% agarose gel electrophoresis of RT-PCR products for the specific detection of LigCSV RNA1 (primer pair *p31*, expected amplicon size: 123 bp) and LigCSV RNA2 (primer pair *p23*, expected amplicon size: 420 bp) of LigCSV. I: Molecular weight marker, 100 bp DNA Ladder (Promega, Madison, WI, United States); II: Molecular weight marker, 1 kb DNA Ladder (Promega). Lanes 1–20: Selected symptomatic individual lesions from mite-infested arabidopsis plants; lane 21: Reverse-transcription reaction negative control; lane 22: PCR negative control; and lane 23: LigCSV-infected *Ligustrum japonicum* plant, positive control of the RT-PCR tests.

*Brevipalpus* mites from the privet sample SPa1 were transferred to healthy arabidopsis plants. Seven to ten days after infestation, leaves showing yellow spots, likely symptoms of the infection, were collected (Figure 6A). RT-PCR tests using specific primers detected the presence of LigCSV in 22 leaf samples (Figure 6B). Mites found after leaf collection were kept in ethanol solution for further identification.

### Mites in the Infected Plants Belong to Three Species of the Genus *Brevipalpus*

Flat mites collected from field samples and arabidopsis plants used in the transmission experiments were morphoanatomically examined. Analyzed mites were distributed as follows: sample Cdb1, field-collected mites: 79; sample Prb1, field-collected mites: 407, after transmission experiments: 50; sample SPa1, field-collected mites: 25, after transmission experiments: 15; and sample Crb1, field-collected mites: 54.

**FIGURE 7:**
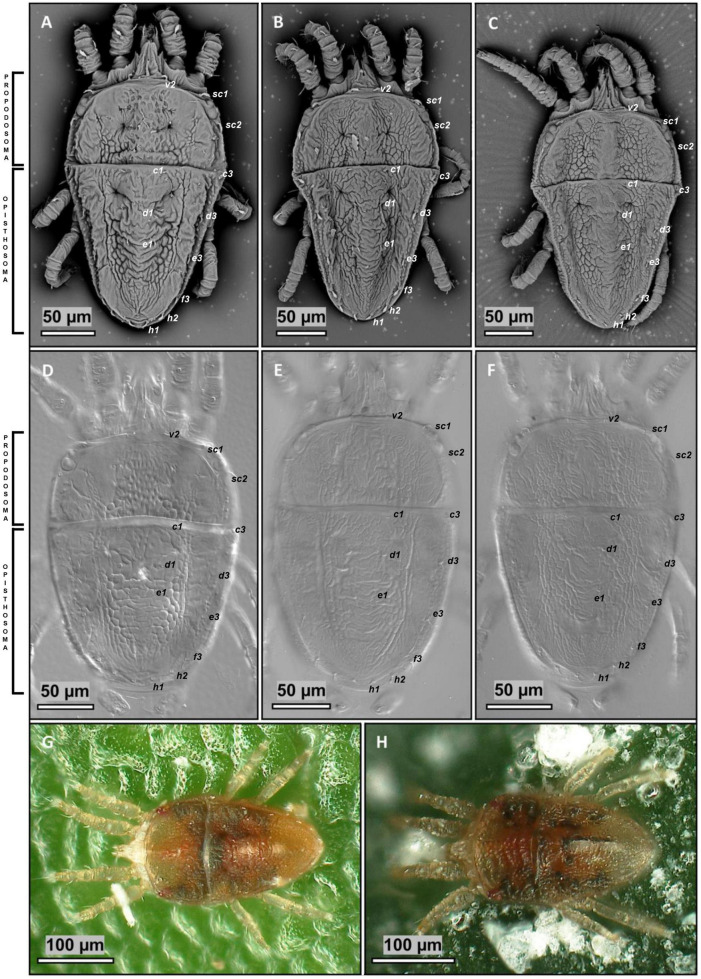
Morphoanatomical identification of *Brevipalpus* mites. **(A,B,H)** *Brevipalpus papayensis* mite collected from *Ligustrum* spp. plants, samples Crb1 and SPa1, **(B,E)** *Brevipalpus tucuman* collected from *Ligustrum sinense* plant, sample Cdb1. **(C**,**F**,**G)** *Brevipalpus obovatus* gathered from *Solanum violifolium* plant, sample Prb1. **(A–C)** Scanning electron microscopy (SEM) micrographs of the cuticle of the mite dorsum. **(D–F)** Differential interference contrast (DIC) micrographs of dorsal view of the reticulation pattern. **(G,H)** Stereoscopic light photographs.

Mites from samples Crb1 and SPa1 were assigned to *B. papayensis* (Figures 7A,D,H), whereas those from the sample Cdb1 to *B. tucuman* (Figures 7B,E), and from sample Prb1 to *B. obovatus* (Figures 7C,F,G). The following traits were observed: (i) dorsal ophistosoma with six setae, *f2* absent, and palps four segmented, with one solenidion and two eupathidia on distal segment on mites of the three species; (ii) presence of two solenidia on tarsus II in *B. papayensis* and *B. tucuman*, and one solenidium in *B. obovatus*; (iii) prodosum with central cuticle with broad areolae on *B. papayensis* and *B. tucuman* and smooth on *B. obovatus*; (iv) in the dorsal opisthosoma, *d1-d1* to *e1-e1* weakly reticulate anteriorly, and short transverse fold posterior to *e1* on *B. obovatus*, weakly wrinkled on the anterior part, transversal folds becoming longitudinal folds toward *h1* on *B. papayensis*, and with some folds and wrinkles anteriorly, few transverse folds between *d1-d1* to *e1-e1*, with transverse folds posteriorly on *B. tucuman*; (v) ventral plate with small rounded cells becoming fused centrally on *B. obovatus*, bands of mixed orientations on *B. papayensis*, and irregularly shaped cells on *B. tucuman* (data not shown); (vi) spermathecal apparatus ending in small tick vesicle covered on its entire surface with small projection on *B. obovatus*, big spherical vesicle with projection all around the surface on *B. papayensis*, and rounded vesicle similar in size with *B. papayensis* covered with tiny projections on *B. tucuman* (data not shown).

### Solanum Violifolium Ringspot Virus, Ligustrum Chlorotic Spot Virus, and Ligustrum Leprosis Virus Show Bacilliform, Enveloped Virion Particles of Different Sizes

**FIGURE 8:**
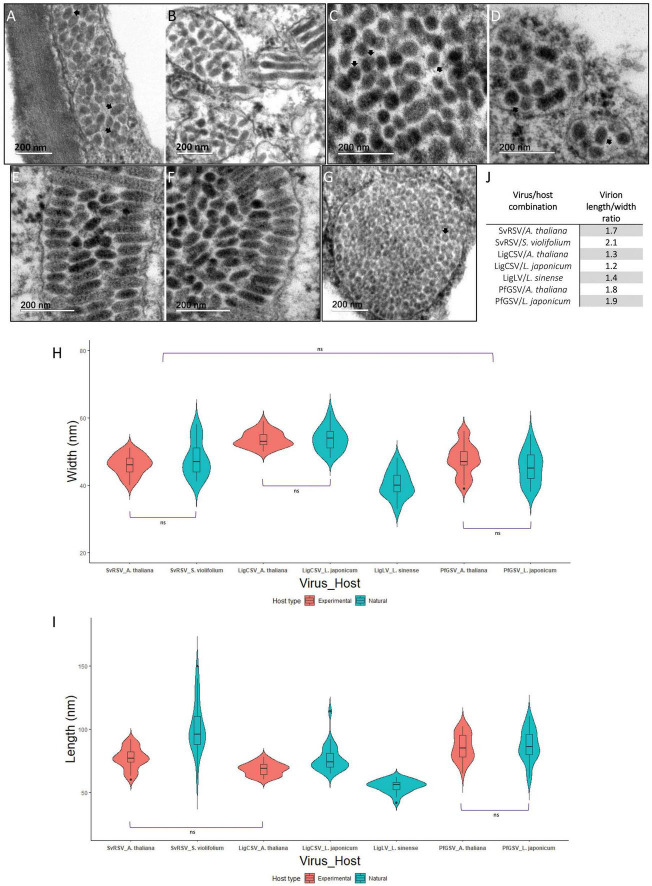
Size and morphology of virions from SvRSV, LigCSV, and LigLV. Microphotographs of ultrathin sections of plants infected by SvRSV_Prb1, LigCSV_SPa1, and LigLV_Cdb1. **(A,B)** SvRSV in *Arabidopsis thaliana* and *Solanum violifolium* plants, respectively. **(C,D)** LigCSV in *A. thaliana* and *Ligustrum japonicum* plants. **(E,F)** PfGSV in *A. thaliana* and *L. japonicum* plants. **(G)** LigLV in *L. sinense* plant. **(H,I)** Violin plots represent the width and length of 25 viral particles measured in at least three micrographs of each virus/host combination. Values of each group were compared using the Wilcoxon signed-rank test. Horizontal brackets depict the results of the statistical test. ns: non-significant difference. In all other comparisons, significant differences were observed (*p* < 0.05). A detailed description of statistical analysis results is described in Supplementary Tables 4, 5. **(J)** Length/width ratio of virions from SvRSV, LigCSV, LigLV, and PfGSV in natural and experimental plant hosts.

Preliminary examination by TEM of ultrathin sections of plant tissues revealed differences in width and length among particles from SvRSV, LigCSV, and LigLV (Figure 2). To get insight into virion dimensions, 25 particles of the three viruses were measured from TEM microphotographs taken at the same magnification (200,000×) (Figures 8A–G). The diameter and length of virions of SvRSV and LigCSV were assessed in particles detected in both their natural hosts and arabidopsis plants. For the sake of comparisons, the same evaluations were carried out with virions of the typical cilevirus PfGSV in ultrathin sections of arabidopsis and *L. japonicum* plants. In the case of LigLV_Cdb1, analyses were circumscribed to its natural host, *L. sinense*.

Regardless of the analyzed host, virions from SvRSV and PfGSV had a similar width, approximately 45–46 nm (*p* < 0.05), but they were wider than those from LigLV (40.3 ± 4.0 nm) and narrower than particles from LigCSV in arabidopsis (53.6 ± 2.3 nm) and *L. japonicum* (53.8 ± 3.6 nm) (*p* < 0.05) (Figure 8H and Supplementary Table 4). In mean values, virions of SvRSV and LigCSV observed in arabidopsis plants were shorter than those observed in their corresponding natural hosts (Figure 8I). Although uncommon, virus particles of up to 120–150 nm long were observed in *S. violifolium* and *L. japonicum* plants infected by SvRSV and LigCSV, respectively (Figures 8B,D), and their mean length values were 101.6 ± 20.7 nm and 65.9 ± 10.6 nm, respectively. With a mean length of 55.0 ± 4.5 nm, virions of LigLV were the shortest particles evaluated in this study (Supplementary Table 5). Virions of PfGSV infecting both *L. japonicum* and arabidopsis had the same length, approximately 86–87 nm (Figures 8E,F).

Detailed analyses of ultrathin sections of the infected plants also revealed the presence of an outer membranous layer surrounding the particles of the three studied viruses (Figures 8A,C,D,G). These structures were best distinguished during the observation directly in the TEM.

### Detection of Mixed Infection of Solanum Violifolium Ringspot Virus and Ligustrum Chlorotic Spot Virus

Both HTS and RT-PCR tests revealed the presence of mixed infection by SvRSV and LigCSV in the sample Crb1. Detailed analysis of the relative abundance of reads corresponding to these two viruses in the HTS library suggested that SvRSV viral load was greater than that of LigCSV (Table 2). In addition, in the case of LigCSV, RNA1-derived reads were almost sixfold more abundant than those from its RNA2. To best characterize the distribution of viral genomic molecules in the sample Crb1, the presence of RNA1 and RNA2 molecules of these two viruses were tested in individual lesions by RT-PCR.

**FIGURE 9:**
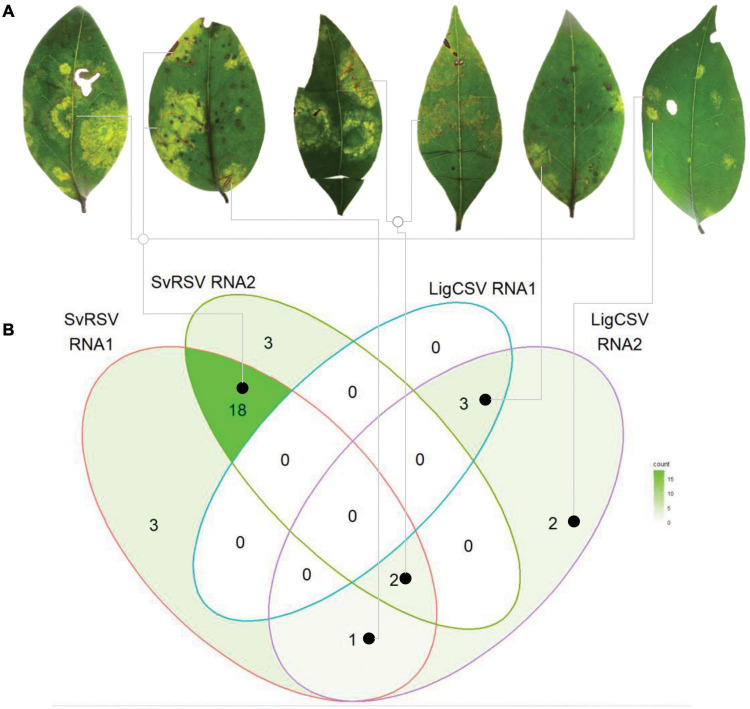
Distribution of SvRSV and LigCSV in isolated lesions found in leaves of *Ligustrum lucidum* plants of the sample Crb1. **(A)** Leaf discs comprising viral symptoms were collected and independently processed for the specific detection of RNA1 and RNA2 of each virus. **(B)** The Venn diagram represents the general distribution of the detected viral genome RNA molecules across the 54 analyzed lesions. Gray lines link lesions to the viral RNA detection indicated in the diagram.

A total of 54 randomly selected lesions including spots of different sizes and morphologies, e.g., absence or presence of necrotic tissues, yellowish blotches, or markedly chlorotic spots, were independently analyzed (Figure 9A). Viral RNAs were detected in 32 (59%) of the lesions. Single infection by SvRSV was most common, with SvRSV RNA1 + RNA2 detected in 18 of the lesions. Conversely, LigCSV RNA1 + RNA2 alone were only detected in 3 lesions (Figure 9B and Supplementary Figure 2). In some lesions, only one RNA molecule, e.g., RNA1 or RNA2 of SvRSV, or RNA2 of LigCSV, could be detected, but RNA1 of LigCSV was never detected alone. The heterologous combination SvRSV RNA1 + LigCSV RNA2 and the mixed infection SvRSV with the RNA2 of LigCSV were detected in one and two lesions, respectively. Any association between RNA composition and morphology of the lesions could not be observed.

### Non-target Viruses Detected in the High-Throughput Sequencing Libraries

The near-complete sequences of two new narna-like viruses were recovered from samples Prb1 (contigs of 1,670 nts) and Crb1 (contig of 2,462 nts). In addition, the sequence of a novel mitovirus was identified in the sample SPa1 (contig of 2,969 nts). These viruses might have originated from fungi present in the samples. Since non-kitaviruses were outside the scope of this study, further examination was not performed as part of this work.

## Discussion

In this study, we revisited some plant viral diseases in which the molecular identity of their causal agents remained partially characterized or completely unknown (Vergani, 1942; Lima et al., 1991; Ferreira et al., 2007; Kitajima et al., 2010). Leaf samples from four plant species, collected in the southern and southeastern regions of Brazil and Pampas in Argentina, showed chlorotic and/or necrotic spots comparable to those generally found in plants affected by *Brevipalpus*-transmitted viruses (Freitas-Astúa et al., 2018). In addition, flat mites of the genus *Brevipalpus* were recovered from the collected plant samples and in three of these plants, we preliminarily detected fragments of the tentative cilevirus SvRSV by RT-PCR tests using specific primers (Ferreira et al., 2007).

Based on a combined strategy of HTS and conventional Sanger sequencing, analyses of the plant viromes enabled us to recover the genomic sequences of several isolates of three viruses. In agreement with the preliminary RT-PCR tests, two isolates corresponded with SvRSV, while the other ones were assigned to two novel kita-like viruses that we identified as LigCSV and LigLV. Genomic data indicated that the highest nt sequence identity of these viruses always corresponded to genes also found in either one of the other two viruses described in this study or members of the genus *Cilevirus*. Still, the values of nucleotide sequence identities obtained in the comparisons of every viral ORF were below 76%.

Besides the relatively low identity values with other kitaviruses, SvRSV, LigCSV, and LigLV display a distinguishing genomic organization. In comparison with typical cileviruses, the RNA2 segments of SvRSV, LigCSV, and LigLV are shorter, lacking a region of approximately 1.5 kb at the 5′-end of the molecule. In cileviruses, the genomic region upstream of the ORF *p61* includes the ORF *p15* and other small ORFs encoding putative proteins with predicted TM helixes (Ramos-González et al., 2021). In PfGSV, the region also harbors orphan ORFs encoding predicted proteins > 11 kDa of unknown functions (Ramos-González et al., 2020). An RNA2 genomic molecule resembling a 5′-end truncated version of the RNA2 of typical cileviruses was also described in the cile-like virus HYBV (Olmedo-Velarde et al., 2021). However, the information in the RNA2 of HYBV is not completely equivalent to that in the RNA2 of SvRSV, LigCSV, and LigLV. While the RNA2 of these viruses comprise genes that are likely orthologs of the ORFs *p61*, *p32*, and *p24* found in typical cileviruses, HYBV RNA2 has an additional ORF at its 3′-end, an ortholog of *p29.* In typical cileviruses, the ORF *p29* is located at the 3′-end of the RNA1.

HYBV has been suggested as an intermediary link between members of the genera *Cilevirus* and *Higrevirus* (Olmedo-Velarde et al., 2021). While the information encoded by RNA2 of HYBV is more similar to that encoded by RNA2 of typical cileviruses, its RNA1 looks like that of the higrevirus HGSV2. If the same logic is extended to our current analysis, the genomes of SvRSV, LigCSV, and LigLV could be regarded as intermediary arrays between those displayed by HYBV and typical cileviruses. Evolutionarily, SvRSV, LigCSV, and LigLV could be considered descendants of a common ancestor of HYBV and typical cileviruses. This transitional position of SvRSV, LigCSV, and LigLV is also supported by the phylogenetic reconstruction based on the RdRp protein presented in this study. Branches encompassing these viruses are flanked by those of HYBV and Pistachio virus Y (PisVY), both placed in a relatively more basal position, and those of the typical cileviruses. PisVY is a partially characterized virus found in pistachio plants (*Pistacia vera*) of the cultivar Ohadi collected in Rafasnjan, Iran. Since the genome organization of PisVY is similar to that in SvRSV, LigCSV, and LigLV, these four viruses may represent new sublineages of kitaviruses. The relatively large genetic distances among viruses of these sublineages and other members of the family *Kitaviridae* suggest the existence of phylogenetic gaps and reinforce the hypothesis that a great diversity of kitaviruses might remain unidentified. However, while further sampling is likely needed, what is relevant after the inclusion of these viruses in the phylogenetic analysis based on the RdRp protein is their contribution to conciliate the evolutionary history among members of the three accepted genera of kitaviruses. In the RdRp phylogenetic tree presented in this study, kitaviruses are distributed by genera from a single node describing a monophyletic clade supported by more than 80% of the trees sampled in the bootstrap analysis. It should be also noted, however, that in the phylogenetic reconstruction using the P24 protein, blunerviruses clustered in a branch also including nelorpiviruses, centiviruses, aphiglyviruses, and other arthropod-infecting viruses, separately from that with the definitive and tentative members of the genera *Cilevirus* and *Higrevirus*. Subdivision of kitaviruses in two sister branches is likely a consequence of the chimeric origin of genomic segments of kitaviruses, as previously described for blunerviruses (Quito-Avila et al., 2013; Morozov et al., 2020).

It has been theorized that the origin of the 5′-end of the RNA2 in typical cileviruses could have involved its acquisition by horizontal gene transfer from a heterologous source (Ramos-González et al., 2021). The finding of SvRSV, LigCSV, and LigLV could add novel hints for the reconstruction of a plausible evolutionary pathway of cileviruses. While RNA1 molecules of SvRSV, LigCSV, LigLV, and typical cileviruses have similar compositions and likely share an ancestral root, the “truncated” RNA2 of an ancestor of SvRSV, LigCSV, and LigLV could have actively participated as a receptor of the 5′-end region of the RNA2 of current cileviruses.

Virion-like particles identified in plants infected with the blunervirus TPNRBV and the cile-like HYBV have spherical shapes with approximately 85 nm and 50–60 nm in diameter, respectively (Hao et al., 2018; Olmedo-Velarde et al., 2021). Typical cileviruses show enveloped bacilliform virions having 40–70 nm wide and 110–120 nm long (Kitajima et al., 2003; Roy et al., 2013). Detailed analysis of ultrathin sections from infected tissues carried out in this study demonstrated that virions of SvRSV, LigCSV, and LigLV are enveloped bacilliform particles with significant differences in their sizes. Particles of SvRSV and LigLV are narrower than those of LigCSV, and particles of LigLV are shorter than those from SvRSV and LigCSV. Hence, with a lesser length/width ratio (Figure 8J), LigLV and LigCSV particles seem more spherical than those of SvRSV. Particles of SvRSV are shorter than those of PfGSV but they show the same width. Taken together, kitavirus virions seem to represent a spectrum of morphologies ranging from quasi-spherical to typical short bacilliform particles. It should be mentioned, however, that even though a large number of virions were analyzed in more than one host, our work is not free of technical constraints which may produce biased results. Measurement of sizes of enveloped virions in fixed tissues may add novel challenges since these particles can be expanded or retracted depending on cellular physiological conditions in the sample at the time of collection, and/or during its conservation and processing for transmission electron microscopy.

The absence of systemic movement is a central topic in the biology of kitaviruses, therefore, successful viral spread even from two distant points in the same plant leaf depends on their arthropod vectors. Evidence indicates that the transmission of the higrevirus HGSV2 and the cile-like virus HYBV is mediated by mites of the genus *Brevipalpus* (Melzer et al., 2012; Olmedo-Velarde et al., 2021), but the direct involvement of these mites in the transmission of kitaviruses has only been verified for typical cileviruses and SvRSV (Ferreira et al., 2007). In this study, we have demonstrated that mites of the species *B. papayensis* collected from infected *L. japonicum* plants can transmit LigCSV to arabidopsis plants. Besides, morphoanatomical characterization of mites collected in *S. violifolium* plants infected with SvRSV indicated the presence of *B. obovatus*, which is in agreement with previous reports of transmission of this virus by this mite species (Ferreira et al., 2007). Finally, the identification of *B. tucuman* in the *L. sinense* plant infected by LigLV would represent the first evidence of the viral vector activity of this mite species, which, however, needs further confirmatory experiments. More than 300 species of the genus *Brevipalpus* have been described but the number of species with virus vector activity is restricted to less than a dozen (Kitajima and Alberti, 2014; de Lillo et al., 2021). Among them, *B. yothersi* has been identified as the main vector of the typical cileviruses CiLV-C, CiLV-C2, and PfGSV (Roy et al., 2015; Ramos-González et al., 2016; de Lillo et al., 2021). CiLV-C and PfGSV can be also vectored by *B. papayensis* (Nunes et al., 2018; Tassi, 2018), which according to the results obtained in this study can also vector LigCSV. Furthermore, since only *B. papayensis* mites were identified in the sample Crb1, infected by LigCSV and SvRSV, our results suggest the transmission of SvRSV by these mites. However, this finding may also emphasize that a wider number of mites from the sample Crb1 need to be analyzed to detect any *B. obovatus* specimen present, which may be in lower frequency. Anyway, the possibility that SvRSV can be also transmitted by *B. papayensis* cannot be ruled out. Both *B. yothersi* and *B. papayensis* have been demonstrated as vectors of more than one species of cilevirus and one species of the genus *Dichorhavirus*, family *Rhabdoviridae* (Chabi-Jesus et al., 2018; de Lillo et al., 2021).

In addition to the mites involved in viral transmission, the examination of *L. lucidum* sample Crb1, where SvRSV and LigCSV were detected, also suggested some clues about kitavirus ecology that will require further study before conclusions can be drawn. Particularly, the use of more sensitive methods would be necessary to detect the presence, if any, of some genomic segments, perhaps at very low concentrations in some lesions, and undetected using conventional RT-PCR tests. Nonetheless, the analysis of the isolated lesions showed (*i*) the predominance of infection loci where only SvRSV could be detected, (*ii*) the lack of lesions with the four genomic RNAs, and (*iii*) the existence, although in low frequency, of heterodox mixes of genomic RNAs, e.g., RNA1 and RNA2 of SvRSV + RNA2 of LigCSV. These findings could be only a reflection of the best transmission efficiency of SvRSV, the highest loads of SvRSV in *L. lucidum*, or the widest distribution and prevalence of SvRSV in that sample. Conversely, although under different experimental conditions, the isolate SPa1 of LigCSV seemed to be efficiently transmitted by *B. papayensis* to arabidopsis. In the experiment with the isolate SPa1, the virus was acquired from a different host, i.e., *L. japonicum*, and particularly, it was in a single infection. Altogether, the snapshot obtained from the sample Crb1 may suggest the existence of undescribed interspecific interaction between kitaviruses in both plants and mites during mixed infections including, perhaps, genomic rearrangements. Mixed infections between a cilevirus and a dichorhavirus infecting citrus have been reported (Roy et al., 2014) but among kitaviruses, only mixed infections of two strains of the same viral species have been described (Chabi-Jesus et al., 2021).

The molecular and biological characterization of SvRSV, LigCSV, and LigLV has also exposed some data and experimental results that might be used as the basis for future analyses. First, the existence of extra-large virions detected in the ultrathin sections of plant tissues infected by SvRSV and LigCSV. In SvRSV, the existence of these particles seems to be an intrinsic feature, since they were also described in previous studies (Ferreira et al., 2007). Indeed, morphological differences between virions of SvRSV and other cileviruses were suggested as evidence supporting its classification in a separated taxon (Ferreira et al., 2007). Whether the atypical virions correspond to a viral species not detected by our virome analysis or they result from defective maturation processes, and whether they have any role in the replication process, are aspects worth studying. Second, a thorough examination of orthologous genes among HYBV, PisVY, typical cileviruses, and the isolates of the three viruses described in this study reveals a common trend to the presence of larger *p29*, *p32*, and *p24* orthologous ORFs in PisVY or HYBV, mid-sized versions of these ORFs in SvRSV, LigCSV or LigLV, and short versions in typical cileviruses (Supplementary Table 6). Finally, the high frequency of small ORFs found in the genome of these viruses and particularly, the large proportion of them that potentially encode peptides with predicted TM helices. Since genome size reduction and the presence of overlapping genes and TM small proteins might be linked and represent central elements to understand processes such as gene expression regulation and virus-host interaction (Belshaw et al., 2007; Wu et al., 2020), we speculate they could be valuable attributes to study the evolutionary process of viruses of the family *Kitaviridae*.

Currently, viruses of the family *Kitaviridae* are assigned to one of three genera according to the number of genomic segments and their genomic organization. Within the genus *Cilevirus*, demarcation of new species ponders the extent of the serological relationship, proteome with less than 85% amino acid sequence identity with other members, natural and experimental host range reactions, and vector species and transmission (Quito-Avila et al., 2021). Based on their genome organization, it seems evident that SvRSV, LigCSV, and LigLV best fit in the genus *Cilevirus*, but considering the relatively low identity between their proteins and the distinct virion morphology of LigCSV and LigLV, it is tentative to speculate that these viruses may require a new taxonomic accommodation. While expecting for the analyses of new kitaviruses that will likely allow getting a better insight into the phylogenetic relationship of kitaviruses, we propose that the viruses described in this study be considered tentative members of the genus *Cilevirus*.

## Data Availability Statement

The virus sequences derived from this study can be found in GenBank under the accession numbers: SvRSV_Prb1 (OK626439 and OK626440), SvRSV_Crb1 (OK626441 and OK626442), LigCSV_SPa1 (OK626447 and OK626448), LigCSV_Crb1 (OK626449 and OK626450), and LigLV_Cdb1 (OK626451 and OK626452).

## Author Contributions

PR-G, CC-J, and AT: conceptualization. PR-G, CC-J, AT, RFC, and EK: formal analysis. RH, EK, and JF-A: funding acquisition. PR-G, CC-J, AT, RFC, CN, RH, EK, and JF-A: investigation. PR-G, CC-J, AT, RFC, RH, and EK: methodology. PR-G, EK, and JF-A: supervision. PR-G: writing—original draft. PR-G, CC-J, AT, RFC, EK, and JF-A: writing—review and editing. All authors contributed to the article and approved the submitted version.

## Conflict of Interest

The authors declare that the research was conducted in the absence of any commercial or financial relationships that could be construed as a potential conflict of interest.

## Publisher’s Note

All claims expressed in this article are solely those of the authors and do not necessarily represent those of their affiliated organizations, or those of the publisher, the editors and the reviewers. Any product that may be evaluated in this article, or claim that may be made by its manufacturer, is not guaranteed or endorsed by the publisher.

## Footnotes

1
https://usegalaxy.org

2
https://www.ncbi.nlm.nih.gov/labs/virus/vssi/#/

3
https://www.ncbi.nlm.nih.gov/orffinder/

4
http://www.cbs.dtu.dk/services/SignalP/

5
https://www.genome.jp/tools/motif/

6
http://www.cbs.dtu.dk/services/TMHMM/

7
http://www.cbs.dtu.dk/services/DeepLoc-1.0/index.php

8
https://www.ebi.ac.uk/Tools/msa/mafft/

9
https://ngphylogeny.fr/

10
http://iqtree.cibiv.univie.ac.at/
