# Supplementary material for: A Novel Lineage of Cile-Like Viruses Discloses the Phylogenetic Continuum Across the Family Kitaviridae
Source: Front Microbiol. 2022 Mar 28;13:836076. doi: 10.3389/fmicb.2022.836076 (PMC8996159; doi:10.3389/fmicb.2022.836076)
Supplement: Supplementary file 1 [file Data_Sheet_1.PDF]

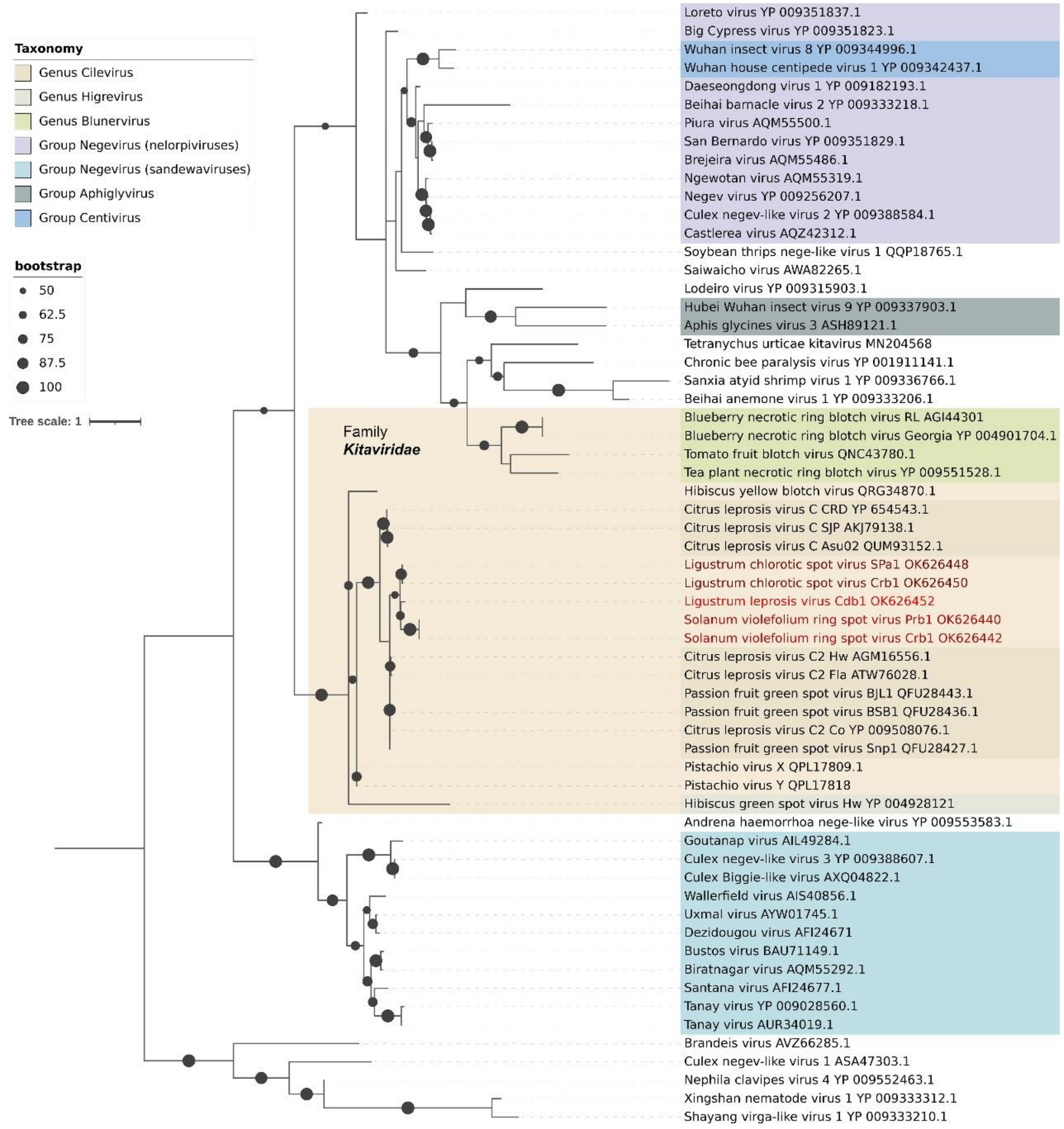

Supplementary Figure 1. Phylogenetic reconstruction for viruses of the family Kitaviridae. Isolates of Solanum violifolium ringspot virus, Ligustrum chlorotic spot virus, and Ligustrum leprosis virus are highlighted in different red tones. The maximum-likelihood phylogenetic tree is based on the deduced amino acid sequences of the P24 protein. Phylogenetic informative regions of the multiple sequence alignment included 58 residues that were selected using BMGE software (Criscuolo and Gribaldo, 2010) and its evolutionary history was inferred based on the model mtZOA+F+G4 chosen according to Bayesian Information Criterion. The bootstrap support values (1,000 replications) of branches greater than 50% are indicated with solid black circles next to the corresponding nodes. The scale bar specifies the average number of amino acid substitutions per site.
