# Supplementary material for: A Novel Lineage of Cile-Like Viruses Discloses the Phylogenetic Continuum Across the Family Kitaviridae
Source: Front Microbiol. 2022 Mar 28;13:836076. doi: 10.3389/fmicb.2022.836076 (PMC8996159; doi:10.3389/fmicb.2022.836076)
Supplement: Supplementary file 2 [file Data_Sheet_2.PDF]

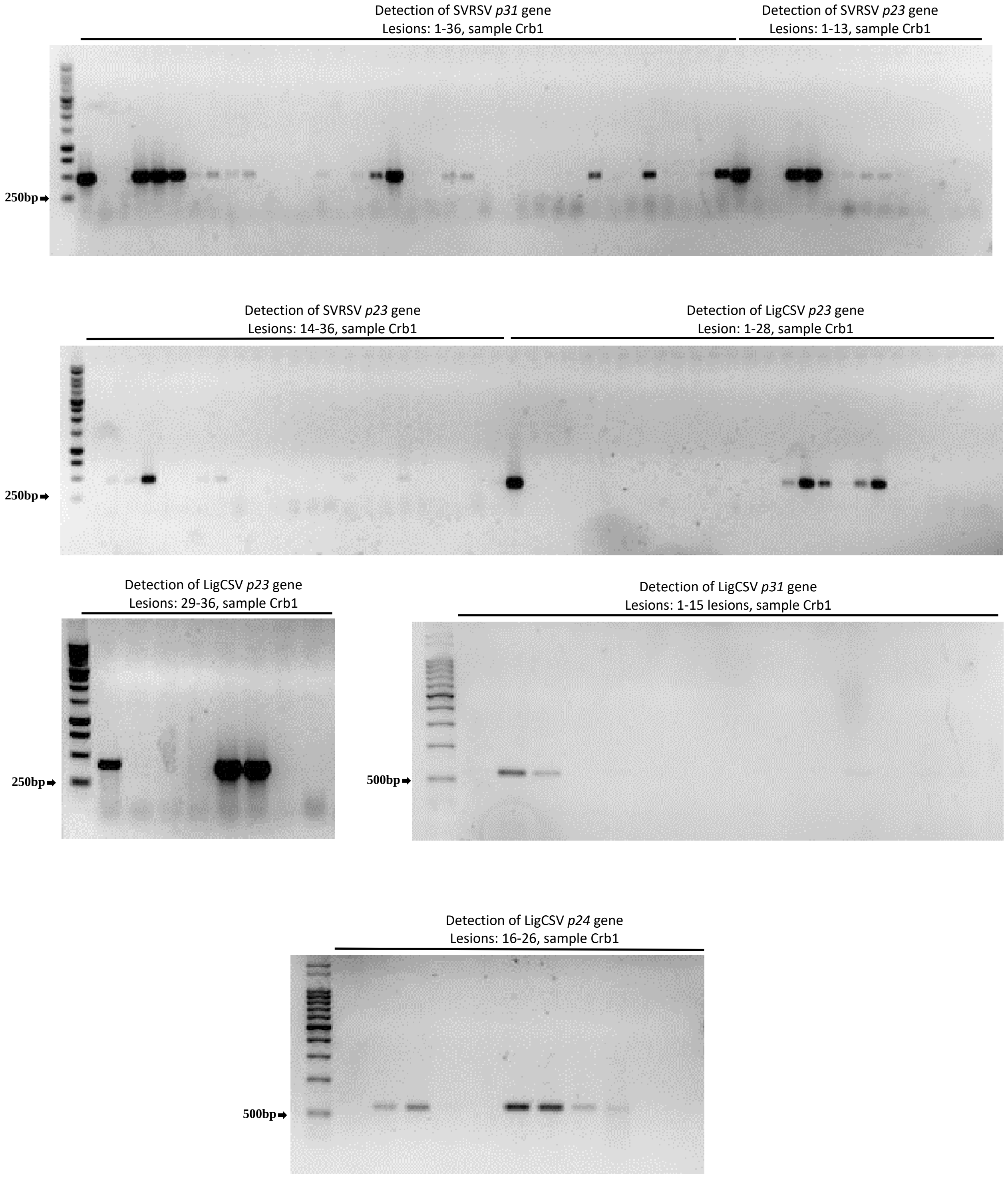

Supplementary Figure 2. Detection of SvRSV and LigCSV in individual lesions of the sample Crb1. Viruses were detected by RT-PCR using specific primers for genes p31 in the RNA1 and p23 in the RNA2 of SvRSV and LigCSV. 1% agarose gel electrophoresis of PCR reaction products of the first 36 samples out of 54 analyzed lesions. The specific detection of RNA1 was carried out using the primer pair p31 SvRSV (expected amplicon size: 490 bp), and p31 LigCSV (expected amplicon size: 123 bp). For the detection of RNA2 were used the primer pairs p23 SvRSV (expected amplicon size: 474 bp) and p23 LigCSV (expected amplicon size: 420 bp). Two molecular weight markers were used: 1kb DNA Ladder (Promega, Madison, WI, USA) and 100bp DNA Ladder (Promega).
