# Supplementary material for: A Novel Lineage of Cile-Like Viruses Discloses the Phylogenetic Continuum Across the Family Kitaviridae
Source: Front Microbiol. 2022 Mar 28;13:836076. doi: 10.3389/fmicb.2022.836076 (PMC8996159; doi:10.3389/fmicb.2022.836076)
Supplement: Supplementary file 3 [file Data_Sheet_3.docx]

**Supplementary Table 1**. Complete list of primers used for resequencing, RACE analyses, and detection of SvRSV (**A**), LigCSV (**B**), and LigLV (**C**). To obtain some genomic fragments, more than one forward (F and Fa) or reverse (R, Ra, and Rb) primers were developed.

(**A**)

| **Virus** | **Primer name**^a^ | **Primer sequence** (5′－3′) | **Target region**^b^ | **Amplicon size** (bp) |
| --- | --- | --- | --- | --- |
| SvRSV | RNA1 | | | |
|  | 1F | ATTTGAATCCTATTTGAC | 390-407 | 860 |
|  | 1R | ACTCGTAAGTATGTAGGG | 1232-1249 |  |
|  | 2F | CTTATTATAGAGGTTTTCC | 1138-1156 | 867 |
|  | 2R | GTTATATGATTCCATATCC | 1986-2004 |  |
|  | 3F | CAGATTTCAGTACTGTTTA | 1811-1829 | 996 |
|  | 3R | CAGTAGACATAATACAAAAA | 2787-2806 |  |
|  | 4F | TGTAGACATAGTTGTTACAC | 2691-2710 | 906 |
|  | 4R | ACTATAATATCCGTCATCT | 3578-3596 |  |
|  | 5F | TAATATCTGTTATCATTGC | 3465-3483 | 959 |
|  | 5R | CGGTAGAAACTAGAGAAT | 4406-4423 |  |
|  | 6F | AGAAAAAGACTAGGAATAA | 4292-310 | 901 |
|  | 6R | GGAAATATATCTCTAAGGA | 5174-5192 |  |
|  | 7F | CAGGAGATTTATTCTAAGTA | 5050-5069 | 885  848a |
|  | 7R | ATCATCATAGACAGGTTC | 5917-5934 |  |
|  | 7Ra | TGTTCTATCCCGTTTTTCTC | 5878-5897 |  |
|  | 8F | GACTGTTTTAGACAAGTTT | 5751-5769 | 563  606a |
|  | 8Fa | ATGGTAAGGTCAACACATTT | 5708-5727 |  |
|  | 8R | TAATATAATCCGAAACAC | 6296-6313 |  |
|  | 9F | ATAACCTTAATTATATGGAC | 6071-6090 | 820 |
|  | 9R | CTATCTCTGACTGAAGATT | 6872-6890 |  |
|  | 10F | TAGAGTTTTGAAAGAAGCCG | 7314-7333 | 713 |
|  | 10R | TAGACAGTTCTCCTAATGCC | 8007-8026 |  |
|  | *p31*_F | CACGTCGTTCAGCAGAA | 7862-7878 | 490 |
|  | *p31*_R | ACCTCTTGGTCATCGACT | 8334-8351 |  |
|  | RNA2 | | | |
|  | 1F | TGACTGGATAGAATGGATCTG | 182-202 | 920 |
|  | 1R | TAAATAGACCTAGCAACATAGGC | 1074-1101 |  |
|  | 2F | CGTATTCACGATGATCTTTAC | 1004-1024 | 1023 |
|  | 2R | TGTCAAAAACCCTATTCCAG | 2007-2026 |  |
|  | 3F | TTGATCAAAGTGAAGTTA | 2655-2672 | 701  1397a |
|  | 3Fa | GTATTTTCAAGCATTCTA | 1959-1976 |  |
|  | 3R | GAACTCAAGGTACTAGAAG | 3337-3355 |  |
|  | RNA1. 5’ RACE | | | |
|  | 5’_RNA1 | GATTACGCCAAGCTTGCGCGCGTTGCGTTAGCAGCTATCATG | 858-884 | - |
|  | 5’_RNA1_ nested | CGTGAAACCGGGTTATC | 486-502 | - |
|  | RNA2. 5’ RACE | | | |
|  | 5’_RNA2 | GATTACGCCAAGCTTACTACAGCGCGACAACGAGGCTGG | 434-457 | - |
|  | 5’_RNA2_ nested | TTCATCGTGGATTGGTTC | 248-265 | - |

| **Virus** | **Primer name**^a^ | **Primer sequence (5′－3′)** | **Target region**^b^ | **Amplicon size** (bp) |
| --- | --- | --- | --- | --- |
| LigCSV | RNA1 | | | |
|  | 1Fa | AAAATTGTTACGTCAAGGCA | 503-522 | 546  774a |
|  | 1R | CAATTGTAACGGGTCTCATC | 1029-1048 |  |
|  | 1Ra | GTGTGGCTTCAAATTTTTGA | 1257-1276 |  |
|  | 2F | TGCTAACCTTTAATATACCTCG | 911-932 | 899  1027a |
|  | 2R | GCCTTTGTACAAACAAGCT | 1791-1809 |  |
|  | 2Ra | TTACCATATACTCCGTCACG | 1913-1932 |  |
|  | 3F | AATAGTAAGAGCCTGCGAG | 1627-1645 | 1885  1799a |
|  | 3Fa | TGACTCTATATGTGAGTGTCG | 1713-1733 |  |
|  | 3R | CATTCCATCTTTCACGACAT | 3492-3509 |  |
|  | 4F | GATTTCATCGCAATTCTATCATT | 3307-3329 | 898 |
|  | 4R | ACGCATTCTTACTAAGGACT | 4185-4204 |  |
|  | 5F | CGCTAGGTTGATGGAAATG | 4117-4035 | 890 |
|  | 5R | TTAAAGTTGGTTGCATGGTT | 4887-4906 |  |
|  | 6F | TCTGGAGAAGTTGAGTAGATT | 4740-4760 | 594 |
|  | 6R | TACCTACTAGGGACAGTAGC | 5314-5333 |  |
|  | 7F | TGCAGATAAGAGCATTTTTAATG | 5139-5161 | 915 |
|  | 7R | GACACACCACTTGATAGTTG | 6034-6053 |  |
|  | 8F | TAATCTAGATCTGGATTTGCAAT | 5856-5878 | 908 |
|  | 8R | CTAACTTCGACAAATCAAAGTT | 6742-6763 |  |
|  | 9F | TCCTGAGGAACTTGTTAAGA | 6582-6601 | 850 |
|  | 9R | TTTTAGACTTCAACGCCTTC | 7412-7431 |  |
|  | 10F | TGGTTACCGTTACTTTTTCTC | 7309-7329 | 830  919a |
|  | 10R | GTTGACATCATCGACATCG | 8120-8138 |  |
|  | 10Ra | AGTAGTGAGGGAAAGAAGTG | 8208-8227 |  |
|  | *p31*_F | GGTCACGAATTATAAGGCAG | 7670-7689 | 373 |
|  | *p31*_R | TGGAATGGCTTTGATAGTCT | 8023-8042 |  |
|  | RNA2 | | | |
|  | 1F | GACGCCTATAGCAGTCATC | 61-79 | 655 |
|  | 1R | AGTAGTGTATCTCCGCTGATT | 695-715 |  |
|  | 2F | CACCTCTCTGAACGTGAGTA | 535-554 | 652 |
|  | 2R | GTCTTCAAAAACTCGAAGGT | 1167-1186 |  |
|  | 3F | GTCCCTTCACGGAATATG | 1037-1054 | 651  702a |
|  | 3Fa | AATTTCCCGTTCACCCATTT | 986-1005 |  |
|  | 3R | ACCTACGTCGATGAGAAAGT | 1668-1687 |  |
|  | 4F | TTAAACCAGAGTCAGAGTGGT | 1551-1571 | 659 |
|  | 4R | TAACGTCAAAGTCAAGTTCG | 2190-2209 |  |
|  | 5F | CCTAATGATCTTGTTTCACG | 2057-2076 | 657 |
|  | 5R | AGTCGATTGACTTCGTTCTT | 2694-2713 |  |
|  | 6F | AAGATCTCGCTAAGGTGGT | 2535-2553 | 676 |
|  | 6R | TCGCATAAATGTGAAGTGAT | 3191-3210 |  |
|  | 7F | CTGGGCAAAGAATAACTTTT | 3029-3048 | 509  420a |
|  | 7F^a^ | TCGGATTGATTGTCTCTGTG | 3118-3137 |  |
|  | 7R | AAACCGGATTTGAATTATATG | 3517-3537 |  |
|  | *mp*_F | GGATATACTCTCGAGCGATT | 1957-1976 | 318 |
|  | *mp*-R | CCCATTTCAGAACCAACATT | 2255-2274 |  |
|  | *p23*_F | CTCTCATAAATTGGGCGAAG | 3019-3038 | 304 |
|  | *p23*_R | GGCAACTCCTTGTAAAGTTT | 3303-3322 |  |
|  | RNA1. 5’ RACE | | | |
|  | 5’_RNA1 | GGCGGCAACCATACAATCAACTATGCC | 568-594 | - |
|  | 5’_RNA1_ nested | AGCAGTCAAGTCGTAAGCACTGT | 465-485 | - |
|  | RNA2. 5’ RACE | | | |
|  | 5’_RNA2 | AGTGTATCTCCGCTGATTCGTCTCGT | 687-712 | - |
|  | 5’_RNA2_ nested | GTGCACGGACCCAAACTGACACA | 407-429 | - |

(**B**)

(**C**)

| **Virus** | **Primer name**^a^ | **Primer sequence (5′－3′)** | **Target region**^b^ | **Amplicon size** (bp) |
| --- | --- | --- | --- | --- |
| LigLV | RNA1 | | | |
|  | 1F | CTCCTAAAGAGTCTTCCGTT | 142-161 | 861 |
|  | 1R | CAGCAACCATACAATCAACA | 983-1002 |  |
|  | 2F | ATATGCGCATAAATGTCCAC | 818-837 | 876 |
|  | 2R | AGACTCTGAGTAGCCTCATA | 1674-1693 |  |
|  | 3F | GAGGAATTGGAATAAGTGCC | 1546-1565 | 898 |
|  | 3R | TGCACTTCAATTCGCATAAT | 2424-2443 |  |
|  | 4F | GCTTGCAGCTAATGAAAAAG | 2282-2301 | 862 |
|  | 4R | CGGAATTTCCTCAGGATAGA | 3124-3143 |  |
|  | 5F | GCAGTGTGCTGTTGTATTAT | 2969-2988 | 904 |
|  | 5R | ATTGCACAACGTACAATCAA | 3853-3872 |  |
|  | 6F | CGAGTACGAGTTTGACAAAA | 3673-3692 | 892 |
|  | 6R | TCGGAAACTATTGGTTCAGA | 4545-4564 |  |
|  | 7F | CCACAAGCGTGATAGAATTT | 4391-4410 | 926 |
|  | 7R | AATCAGTGGACTGGTTTTTG | 5297-5316 |  |
|  | 8F | GGCAGATTCCTTTTGTTTCT | 5119-5138 | 906 |
|  | 8R | TAAGAACGTTGGTTTTTCCC | 6005-6024 |  |
|  | 9F | AAACGATATTGTTGTCGACC | 5828-5847 | 919 |
|  | 9R | TTTAGTCTGGAAAAGGACCA | 6727-6746 |  |
|  | 10F | CTTGGCTAAACGATCAGAAA | 6571-6590 | 917 |
|  | 10R | ACCAATAACATCTTCATCAACA | 7466-7487 |  |
|  | 11F | CTGTCGGTAATAGGTGGTAT | 7336-7355 | 904 |
|  | 11R | GACCAACTAAACGAGACTTG | 8220-8239 |  |
|  | 12F | GCTTCACGGTTGTTCTAATT | 8103-8122 | 637  705a |
|  | 12R | GCAATTTTCTGCGATACAAC | 8720-8739 |  |
|  | 12Ra | ACCAGAAATTAACCAGAATTAGG | 8785-8807 |  |
|  | *RdRp*_F | AAAACCCACACTTTCTGATG | 6677-6696 | 303 |
|  | *RdRp*_R | TTGCACTCGAATAACAAGAC | 6960-6979 |  |
|  | *p32*_F | AAATCAGGCTGTTAATGTCG | 7917-7936 | 435 |
|  | *p32*_R | AGGACACGCAAATTCTTATG | 8332-8351 |  |
|  | RNA2 | | | |
|  | 1F | ATGTTGGTCCGTTTAATCTG | 362-381 | 866 |
|  | 1R | GGAACCTTCATTGTTTCCAA | 1208-1227 |  |
|  | 2F | TGGTATGCTTTTTCCCAGTA | 1056-1075 | 831 |
|  | 2R | AAGAATCTATCAGCAGCAGA | 1867-1886 |  |
|  | 3F | GGTTTTATAGTTGACGTGGG | 1696-1715 | 919  2017a  1803b |
|  | 3R | TGGCCAGTAGTTATAAGACC | 2595-2614 |  |
|  | 3Ra | AAGAAAAGGGGAAGACAAGA | 3693-3712 |  |
|  | 3Rb | GAAACAGTAACAAAACCCCA | 3479-3498 |  |
|  | *mp*_F | TTGTCTCTAATGGATCCGAG | 2359-2378 | 391 |
|  | *mp*_R | GCATTTTCATTTACGCTGTC | 27302749 |  |
|  | *p24*_F | CATGTATGTAGCAGTGTTGG | 2964-2983 | 316 |
|  | *p24*_R | GAGAATTCGCGTTATTGGAT | 3260-3279 |  |
|  | RNA1. 5’ RACE | | | |
|  | 5’_RNA1 | AACGGGCAGCTTCGTCAACATCTGG | 414-438 | - |
|  | 5’_RNA1_ nested | ACATCAATGTCTGCAAGGCTGCA | 326-348 | - |
|  | RNA2. 5’ RACE | | | |
|  | 5’_RNA2 | TCCACAACACGTTCAAAGGCTGCGG | 437-461 | - |
|  | 5’_RNA2_ nested | GCAAGAGGCGTGCAACCACCT | 402-422 | - |

^a^When more than one amplicon was obtained, their sizes are indicated following the nomenclature of the used primers; F: forward, R: Reverse. ^b^Data according to the genome of SvRSV isolate Prb1, LigCSV isolate SPa1 and LigLV isolate Cdb1.

**Supplementary Table 2**. Amino acid motifs across the RdRp of viruses SvRSV, LigCSV, LigLV, and other known kitaviruses. Identification was carried out using MOTIF Search (https://www.genome.jp/tools/motif/).

| RdRp  Pfam motif | Cile-like viruses | | | | | Typical cileviruses | | | Higrevirus |
| --- | --- | --- | --- | --- | --- | --- | --- | --- | --- |
|  | SvRSV_Prb1 | LigCSV_SPa1 | LigLV_Cdb1 | HYBV | PisVY | CiLV-C_Crd1 | CiLV-C2_Co | PfGSV-Snp1 | HGSV_2 |
|  | Motif position in the amino acid polypeptide  (Independent *E-value*) | | | | | | | | |
| RdRP_2^1^ | 2016..2445  (4.6e-95) | 1953..2387  (2.5e-89) | 2069..2504  (6.7e-90) | 2149..2586  (5.6e-93) | 2028..2463  (2.7e-89) | 2057..2492  (7e-87) | 2049..2484  (1.5e-90) | 2051..2480  (2.7e-92) | 2189..2617  (4.3e-86) |
| Viral_helicase1^2^ | 1510..1797  (1.5e-19) | 1455..1737  (9.5e-24) | 1572..1853  (2.3e-18) | 1643..1932  (1.6e-18) | 1520..1804  (3.2e-22) | 1558..1840  (1.2e-24) | 1548..1833  (4.7e-23) | 1550..1835  (1.4e-23) | 1672..1956  (1.7e-18) |
| Vmethyltransf^3^ | 112..397  (1.9e-14) | 49..360  (1.8e-17) | 156..473  (2.8e-17) | 156..472  (3.1e-19) | 16..384  (3.7e-20) | 155..518  (3.1e-18) | 132..513  (1.2e-21) | 133..512  (6.9e-22) | 141..524  (2.4e-22) |
| Chropara_Vmeth^4^ | 162..265  (0.072) | - | - | 172..291  (0.031) | 68..172  (0.13) | - | - | - | - |
| UvrD_C_2^5^ | - | 1689..1729  (0.00044) | 1805..1845  (0.055) | 1883..1932  (0.28) | - | 1793..1835  (9.7e-05) | 1785..1831  (0.00064) | 1787..1832  (0.00067) | - |
| FtsJ^6^ | - | 783..943  (0.0019) | 960..1056  (0.0052) | - | 908..964  (0.64) | 988..1047  (0.007) | 985..1044  (0.043) | 936..1042  (0.57) | 979..1076  (0.0059) |
| AAA_12^7^ | - | 1688..1736  (0.0034) | - | 1883..1928  (0.05) | 1732..1802  (0.11) | 1735..1840  (0.015) | 1784..1834  (0.024) | 1786..1836  (0.0031) | - |
| AAA_19^8^ | - | 1451..1571  (0.015) | 1568..1687  (0.037) | 1638..1757  (0.17) | - | 1558..1674  (0.13) | - | - | - |
| AAA_30^9^ | 1504..1621  (0.0059) | 1450..1571  (0.018) | 1568..1687  (0.038) | - | 1512..1635  (0.00074) | 1555..1674  (0.007) | 1545..1667  (0.0014) | 1547..1669  (0.00061) | - |
| RdRP_3^10^ | - | 2216..2282  (0.035) | 2331..2398  (0.0038) | 2365..2479  (0.0099) | 2243..2358  (0.033) | 2319..2386  (0.012) | - | - | 2453..2519  (0.00097) |
| OTU^11^ | - | 584..677  (0.29) | 697..790  (0.0011) | - | 568..661  (0.19) | 689..777  (0.1) | - | 685..776  (0.036) | - |
| DUF5488^12^ | - | - | 631..658  (0.62) | - | - | - | - | - | - |
| UxaE^13^ | - | - | - | - | 587..686  (0.024) | - | - | - | - |
| G-gamma^14^ | - | - | - | - | - | - | - | 1403..1432  (0.25) | - |
| GMP_synt_C^15^ | - | - | - | - | - | - | - | - | 1454..1505  (0.42) |

^1^PF00978: RNA dependent RNA polymerase, ^2^PF01443: Viral (Superfamily 1) RNA helicase, ^3^PF01660: Viral methyltransferase, ^4^PF19223: Chroparavirus methyltransferase, ^5^PF13538: UvrD-like helicase C-terminal domain, ^6^PF01728: FtsJ-like methyltransferase, ^7^PF13087: AAA domain, ^8^PF13245: AAA domain, ^9^PF13604: AAA domain, ^10^PF00998: Viral RNA dependent RNA polymerase, ^11^PF02338: OTU-like cysteine protease, ^12^PF17590: Family of unknown function. ^13^PF16257: Tagaturonate epimerase, ^14^ PF00631: GGL domain, ^15^PF00958: GMP synthase C terminal domain.

**Supplementary Table 3**. A detailed description of small ORFs detected across the genomes of SvRSV, LigCSV, and LigLV.

| Virus/  Genomic segment | ORFan length (nt) | Genomic position | Theoretical pI/Mw (kDa) | Predicted aa sequence | Predicted molecular feature | |
| --- | --- | --- | --- | --- | --- | --- |
| SvRSV_Prb1/RNA1 | ORF4  (315) | 7605-7919 | 12.90 / 12.36 | mkwllfvvvmatlvsklsrprvlnlrnrggskrgslfqlhralrslisshplrlarnrrlwspelrrkflrlrllpllrsvtlnvrsvrghvvqqkkvwstqlpi | SP | Prediction: Signal peptide (Sec/SPI)  Cleavage site between pos. 20 and 21: SRP-RV.  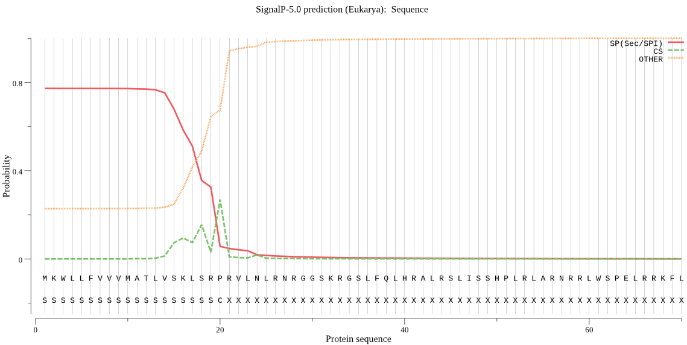 |
| SvRSV_Prb1/RNA2 | ORF4 (231) | 3005-3235 | 8.67 / 9.04 | mwcvgvcpshcrhvgcvpcvhqskcryfpvgnapticfrifclfdrtsrlfqvqahwypcfgctvglcclffvwyfy | TM (1) | 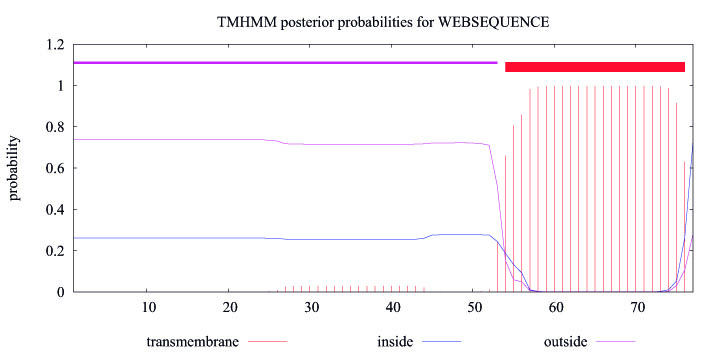 |
| LigCSV_SPa1/RNA1 | ORF2  (333) | 1571-1903 | 10.47 / 12.67 | mtlviilnimsiypiwllqivracemtsqfvifarpkrslnqhtqksmtlyvsvgaksfllwvtviacttallklvctkallcpilslgcsilrssprlllwqlrrmmknl | TM (2) | 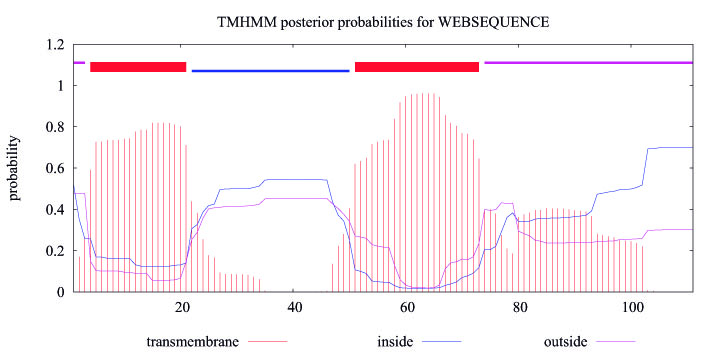 |
|  | ORF3  (288) | 2765-3052 | 9.51 / 11.33 | misnslslrshrlfwwtviwfvqvdyklqlilstcypsvlkvftmymvniftrkyvllnrllrthvicahfilvvvnidhlvfivttdpscptglq | TM (2) | 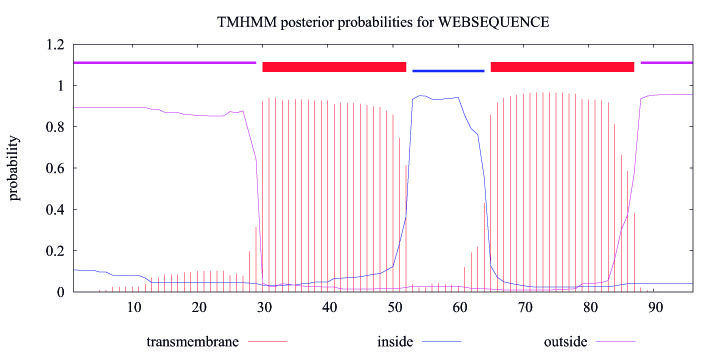 |
| LigCSV_SPa1/RNA2 | ORF1 (201) | 342-542 | 8.89 / 7.38 | mllltsshsimctmvplisvqtvsvwvraqkfpvallqmllsellsvtcsqltitivlgwlktpaph | TM (2) | 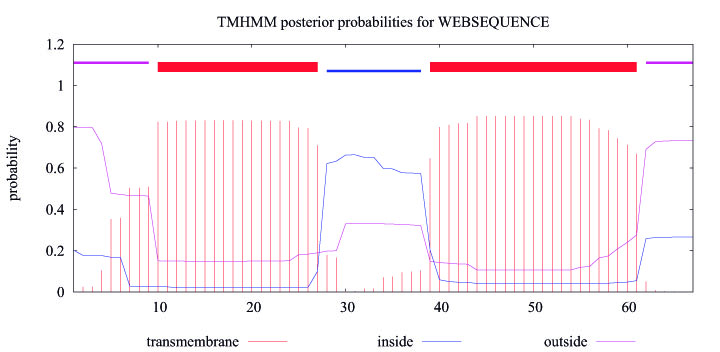 |
| LigLV_Cdb1/RNA1 | ORF1  (144) | 586-729 | 7.66 / 5.57 | myhiimppgfvelfvdtcatllcmivkikyltdtmcllrisvqqctkf | TM | 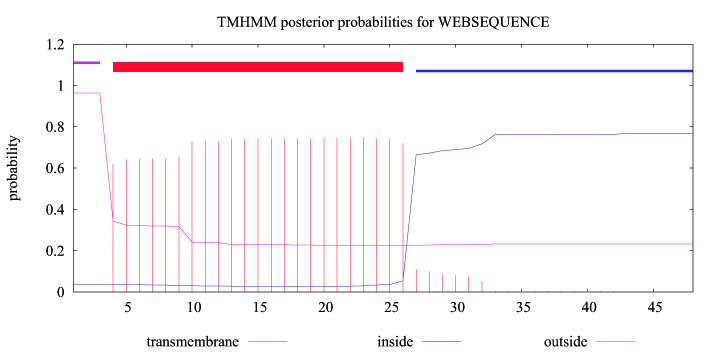 |
| LigLV_Cdb1/RNA2 | ORF1  (108) | 539-646 | 12.30 / 4.38 | mfrrvfmsflvkilviaiiishllgfflrtgfwnhi | TM | 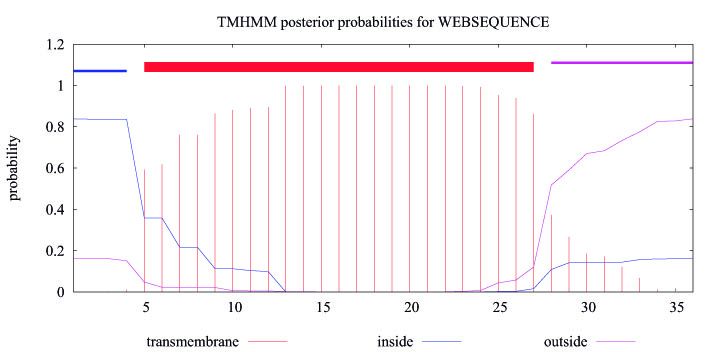 |
|  | ORF3 (285) | 854-1138 | 10.01 / 10.91 | mefnvlmvlirksinspsakiiaflshmmkgygrmsllvlwvftllittfplptfngvyhriilstivvcffpvelvmitiiyvkallfqlakmi | TM (2) | 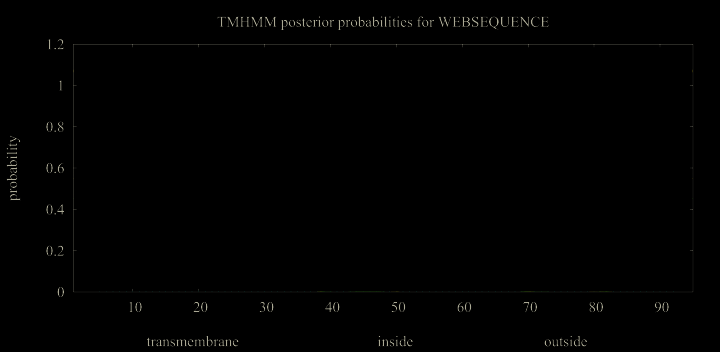 |
|  | ORF4  (144) | 1262-1405 | 9.99 / 5.72 | mimvmlvlystlattfvaiqmvvrivvwttgyfkgpverkyvyrrenf | TM | 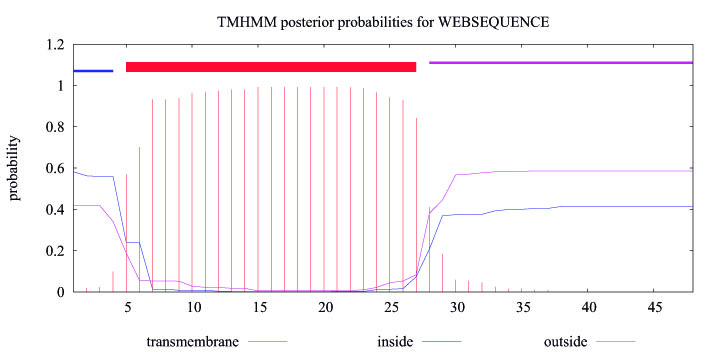 |
|  | ORF7 (300) | 2155-2454 | 11.83 / 11.58 | msnhfdrsqislgsllhrsrlllillflllilailqvrsmirlllsviptvlvslfltalilfrilivlslmdprhiegwlysmrcnvtrlrvlllllil | TM (2) | 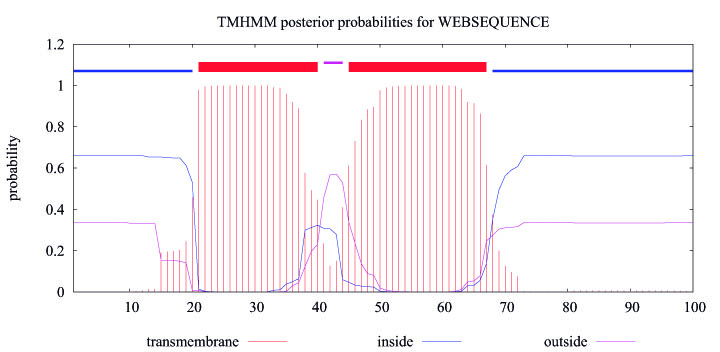 |
|  | ORF10  (147) | 3447-3593 | 7.60 / 5.54 | mwcgvtklvtpgvlllflspltletvlilylvtctvtrcnlcinrdcff | TM | 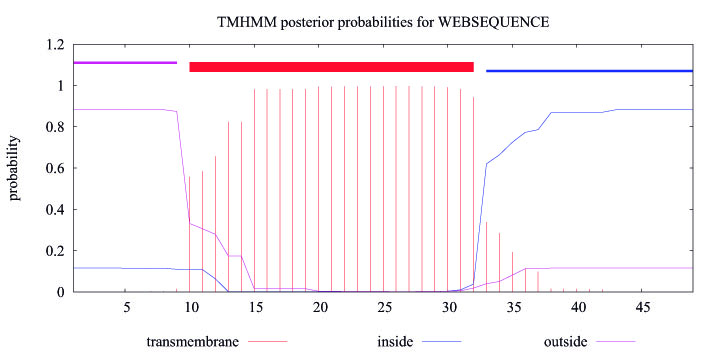 |

**Supplementary Table 4**. Width assessment from virions of SvRSV, LigCSV, and LigLV in natural and experimental hosts.

| **Width by virus and host (nm)** | | | | | | |
| --- | --- | --- | --- | --- | --- | --- |
| SvRSV/  *A. thaliana* | SvRSV/  *S. violifolium* | LigCSV/  *A. thaliana* | LigCSV/  *L. japonicum* | PfGSV/  *A. thaliana* | PfGSV/  *L. japonicum* | LigLV/  *L. sinense* |
| 43  47  46  48  47  50  51  49  45  46  50  44  41  42  49  46  47  40  45  50  47  42  46  44  48 | 41  45  47  55  58  42  44  49  41  57  56  41  46  43  51  53  55  48  47  48  45  43  44  46  48 | 55  57  59  54  57  52  52  51  50  53  56  50  51  52  53  52  53  53  52  55  56  52  54  56  55 | 52  57  55  53  49  48  50  52  50  58  57  54  60  54  52  55  54  51  58  50  56  53  55  49  62 | 41  43  48  47  50  52  56  40  50  39  42  46  55  52  47  46  51  42  46  50  56  49  47  46  49 | 38  40  42  46  45  47  49  43  42  40  51  53  50  55  49  39  38  45  48  47  42  52  39  45  48 | 43  37  46  33  37  42  38  44  39  40  47  42  45  48  38  39  44  33  36  38  41  42  38  37  40 |
| Mean ± SD | | | | | | |
| 46.1 ± 2.9 | 47.7 ± 5.3 | 53.6 ± 2.3 | 53.8 ± 3.6 | 47.6 ± 4.7 | 45.3 ± 4.9 | 40.3 ± 4.0 |

Kruskal-Wallis test by rank. Comparison of multiple groups (non-parametric test)

|  | chi-squared | df | *p* | *p.* adj | *p.* format | p. signif. |
| --- | --- | --- | --- | --- | --- | --- |
| Width | 95.57 | 6 | 2.10e-18 | 2.1e-18 | <2e-16 | **** |

Wilcoxon signed-rank test. Comparison between two groups (non-parametric).

| Virus/Host combination | | *p* | *p.* adj | *p.* format | p. signif. |
| --- | --- | --- | --- | --- | --- |
| SvRSV_*A. thaliana* | SvRSV_*S. violifolium* | 0.53 | 1 | 0.53 | ns |
| SvRSV_*A. thaliana* | LigCSV_*A. thaliana* | 2.64 | 5e-08 | 2.6e-09" | **** |
| SvRSV_*A. thaliana* | LigCSV_*L. japonicum* | 2.39-08 | 4.3e-07 | 2.4e-08 | **** |
| SvRSV_*A. thaliana* | LigLV_*L. sinense* | 6.39e-06 | 8.1e-05 | 6.4e-06 | **** |
| SvRSV_*A. thaliana* | PfGSV_*A. thaliana* | 0.21 | 1 | 0.21562 | ns |
| SvRSV_*A. thaliana* | PfGSV_*L. japonicum* | 0.50 | 1 | 0.50186 | ns |
| SvRSV_*S. violifolium* | LigCSV_*A. thaliana* | 0.00015 | 0.0014 | 0.00015 | *** |
| SvRSV_*S. violifolium* | LigCSV_*L. japonicum* | 0.00012 | 0.0012 | 0.00012 | *** |
| SvRSV_*S. violifolium* | LigLV_*L. sinense* | 6.20e-06 | 8.1e-05 | 6.2e-06 | **** |
| SvRSV_*S. violifolium* | PfGSV_*A. thaliana* | 0.83 | 1 | 0.83817 | ns |
| SvRSV_*S. violifolium* | PfGSV_*L. japonicum* | 0.17 | 1 | 0.17046 | ns |
| LigCSV_*A. thaliana* | LigCSV_*L. japonicum* | 1 | 1 | 1.00000 | ns |
| LigCSV_*A. thaliana* | LigLV_*L. sinense* | 1.31e-09 | 2.8e-08 | 1.3e-09 | **** |
| LigCSV_*A. thaliana* | PfGSV_*A. thaliana* | 5.39e-06 | 7.6e-05 | 5.4e-06 | **** |
| LigCSV_*A. thaliana* | PfGSV_*L. japonicum* | 1.27e-07 | 2.2e-06 | 1.3e-07 | **** |
| LigCSV_*L. japonicum* | LigLV_*L. sinense* | 1.44e-09 | 2.9e-08 | 1.4e-09 | **** |
| LigCSV_*L. japonicum* | PfGSV_*A. thaliana* | 2.43e-05 | 0.00027 | 2.4e-05 | **** |
| LigCSV_*L. japonicum* | PfGSV_*L. japonicum* | 4.35e-07 | 7e-06 | 4.4e-07 | **** |
| LigLV_*L. sinense* | PfGSV_*A. thaliana* | 4.06e-06 | 6.1e-05 | 4.1e-06 | **** |
| LigLV_*L. sinense* | PfGSV_*L. japonicum* | 0.00057 | 0.0046 | 0.00057 | *** |
| PfGSV_*A. thaliana* | PfGSV_*L. japonicum* | 0.11 | 0.77 | 0.11070 | ns |

**Supplementary Table 5**. Length assessment from virions of SvRSV, LigCSV, and LigLV in natural and experimental hosts.

| **Length by virus and host (nm)** | | | | | | |
| --- | --- | --- | --- | --- | --- | --- |
| SvRSV/  *A. thaliana* | SvRSV/  *S. violifolium* | LigCSV/  *A. thaliana* | LigCSV/  *L. japonicum* | PfGSV/  *A. thaliana* | PfGSV/  *L. japonicum* | LigLV/  *L. sinense* |
| 80  74  81  69  74  75  65  63  60  78  65  70  84  86  90  74  92  88  79  82  78  88  75  76  77 | 96  120  87  88  94  60  75  95  110  105  95  92  87  83  130  95  82  140  110  115  98  102  150  132  100 | 62  70  69  72  74  72  64  62  69  69  70  63  69  78  75  71  62  65  68  66  60  72  76  67  62 | 114  87  73  75  67  74  69  70  85  71  81  75  80  68  74  71  72  69  76  92  86  65  86  73  67 | 65  83  76  100  102  96  85  76  78  92  95  100  98  72  76  78  85  86  90  79  85  79  74  102  95 | 80  100  70  80  110  80  90  62  61  83  84  76  78  80  83  86  90  95  100  103  95  96  87  108  100 | 53  62  52  56  57  55  60  55  52  56  50  55  58  49  57  42  46  50  55  59  61  62  58  56  60 |
| Mean ± SD | | | | | | |
| 76.9 ± 8.5 | 101.6 ± 20.7 | 68.3 ± 4.9 | 65.9 ± 10.6 | 85.9 ± 10.6 | 87.1 ± 12.9 | 55.0 ± 4.5 |

Kruskal-Wallis test by rank. Comparison of multiple groups (non-parametric test)

|  | chi-squared | df | *p* | *p.* adj | *p.* format | p. signif. |
| --- | --- | --- | --- | --- | --- | --- |
| Length | 113.26 | 6 | 4.23e-22 | 4.2e-22 | <2e-16 | **** |

Wilcoxon signed-rank test. Comparison between two groups (non-parametric).

| Virus/Host combination | | *p* | *p.* adj | *p.* format | p. signif. |
| --- | --- | --- | --- | --- | --- |
| SvRSV_*A. thaliana* | SvRSV_*S. violifolium* | 1.40515E-06 | 0.000017 | 0.000014 | **** |
| SvRSV_*A. thaliana* | LigCSV_*A. thaliana* | 0.00017647 | 0.0018 | 0.00018 | *** |
| SvRSV_*A. thaliana* | LigCSV_*L. japonicum* | 0.431399916 | 0.86 | 0.43140 | ns |
| SvRSV_*A. thaliana* | LigLV_*L. sinense* | 2.22412E-09 | 0.00000004 | 0.000000022 | **** |
| SvRSV_*A. thaliana* | PfGSV_*A. thaliana* | 0.004999225 | 0.02 | 0.00500 | ** |
| SvRSV_*A. thaliana* | PfGSV_*L. japonicum* | 0.00229189 | 0.014 | 0.00229 | ** |
| SvRSV_*S. violifolium* | LigCSV_*A. thaliana* | 3.12654E-08 | 0.00000047 | 0.00000031 | **** |
| SvRSV_*S. violifolium* | LigCSV_*L. japonicum* | 1.8821E-06 | 0.000021 | 0.000019 | **** |
| SvRSV_*S. violifolium* | LigLV_*L. sinense* | 2.22805E-09 | 0.00000004 | 0.000000022 | **** |
| SvRSV_*S. violifolium* | PfGSV_*A. thaliana* | 0.002778829 | 0.014 | 0.00278 | ** |
| SvRSV_*S. violifolium* | PfGSV_*L. japonicum* | 0.008721366 | 0.026 | 0.00872 | ** |
| LigCSV_*A. thaliana* | LigCSV_*L. japonicum* | 0.000718033 | 0.0065 | 0.00072 | *** |
| LigCSV_*A. thaliana* | LigLV_*L. sinense* | 3.4484E-09 | 0.000000055 | 0.000000034 | **** |
| LigCSV_*A. thaliana* | PfGSV_*A. thaliana* | 5.32846E-08 | 0.00000075 | 0.00000053 | **** |
| LigCSV_*A. thaliana* | PfGSV_*L. japonicum* | 7.25121E-07 | 0.0000094 | 0.0000073 | **** |
| LigCSV_*L. japonicum* | LigLV_*L. sinense* | 1.38153E-09 | 0.000000029 | 0.000000014 | **** |
| LigCSV_*L. japonicum* | PfGSV_*A. thaliana* | 0.001398718 | 0.011 | 0.00140 | ** |
| LigCSV_*L. japonicum* | PfGSV_*L. japonicum* | 0.001950083 | 0.014 | 0.00195 | ** |
| LigLV_*L. sinense* | PfGSV_*A. thaliana* | 1.37404E-09 | 0.000000029 | 0.000000014 | **** |
| LigLV_*L. sinense* | PfGSV_*L. japonicum* | 2.07684E-09 | 0.000000039 | 0.000000021 | **** |
| PfGSV_*A. thaliana* | PfGSV_*L. japonicum* | 0.579638419 | 0.86 | 0.57964 | ns |

**Supplementary Table 6**. Heat map depicting the length variation of ORFs across putative and definitive virus members of the family *Kitaviridae*. Red and green colors indicate the larger and smaller ORFs, respectively, by each column.

| **Virus** | | **Viral ORF orthologues** | | | | |
| --- | --- | --- | --- | --- | --- | --- |
|  |  | *RdRp* | *p29*^1^ | *p61* | *p32* | *p24* |
| Cile-like viruses | PisVY | 7455 | 936 | 1794 | 978 | 612 |
|  | HYBV | 7821 | 936 | 1458 | 903 | 693 |
|  | SvRSV_Pcb1 | 7416 | 846 | 1662 | 897 | 630 |
|  | LigCSV_SPa1 | 7227 | 846 | 1593 | 912 | 621 |
|  | LigLV | 7575 | 882 | 1602 | 921 | 627 |
| Typical cileviruses | CiLV-C_Crd1 | 7539 | 792 | 1614 | 894 | 645 |
|  | CiLV-C_Co | 7515 | 792 | 1635 | 879 | 621 |
|  | PfGSV_Snp1 | 7521 | 777 | 1632 | 888 | 624 |

^1^ORFs have been called according to the CiLV-C genome.
